# Carbon dots penetrating the blood-brain barrier for central nervous system nanomedicine

**DOI:** 10.7150/thno.130380

**Published:** 2026-02-26

**Authors:** Wubshet Mekonnen Girma, Girum Getachew Demissie, Shewaye Lakew Mekuria, Shamsa Kizhepat, T.M. Subrahmanya, Akash S. Rasal, Binyam Abdu Berhe, Gangaraju Gedda, Yoo-Jin Park, Jia-Yaw Chang, Myung-Geol Pang

**Affiliations:** 1 Department of Animal Science & Technology and BET Research Institute, Chung-Ang University, Anseong, Gyeonggi-do 17546, Republic of Korea.; 2 Department of Chemistry, College of Natural Science, Wollo University, P.O. Box 1145, Dessie 1000, Ethiopia.; 3 Nanochemistry Laboratory Center, Department of Chemical Engineering, National Taiwan University of Science and Technology, Taipei 106335, Taiwan.; 4 Department of Chemistry, Faculty of Natural Science, Wolaita Sodo University, Wolaita Sodo, Ethiopia.; 5 Department of Chemistry, College of Natural and Computational Sciences, University of Gondar, Gondar 196, Ethiopia.; 6 Advanced Membrane Materials Research Centre, Graduate Institute of Applied Science and Technology, National Taiwan University of Science and Technology, Taipei 106335, Taiwan.; 7 Department of Chemical Engineering, National Taiwan University, Taipei 106319, Taiwan.

**Keywords:** blood-brain barrier, central nervous system, carbon dots, imaging, therapy

## Abstract

Central nervous system (CNS) diseases are challenging to treat because of the blood-brain barrier (BBB), formed by tight junctions that limit the transcellular transport of therapeutic drugs. Carbon dots (CDs) have emerged as versatile nanotheranostic platforms for the targeting, diagnosis, and treatment of CNS diseases owing to their ultrasmall size, intrinsic photoluminescence, tunable surface chemistry, and biocompatibility. Surface modifications of CDs with targeting ligands, polymer coatings, biomimetic membranes, and exosome-like molecules enable BBB penetration and selective brain accumulation. CDs also support multimodal imaging techniques, such as fluorescence, magnetic resonance, and photoacoustic imaging, for early disease detection and real-time therapeutic monitoring. In addition, their ability to deliver drugs, genes, and therapeutic agents, combined with their antioxidant, anti-inflammatory, photothermal, photodynamic, and sonodynamic properties, highlights their potential for the integrated diagnosis and treatment of CNS diseases. This review systematically summarizes the background of CDs, the design of BBB-penetrating CDs, and their applications in tumor diagnosis, treatment, and imaging-guided cooperative therapies for CNS diseases. Finally, current obstacles and future perspectives are discussed. This review provides a valuable reference for the rational design of BBB-penetrating CDs for the precise treatment of neurological disorders and brain cancers.

## 1. Introduction

Central nervous system (CNS) diseases pose a substantial threat to human health and place a substantial burden on society 1. Although the incidence and mortality rates of peripheral diseases have declined, mortality associated with brain disorders remains elevated. As the population ages, addressing brain diseases has become increasingly critical 2, 3. Alzheimer's disease, Parkinson's disease, epilepsy, schizophrenia, traumatic brain injury, stroke, cerebral ischemia, lysosomal storage diseases, anxiety, depression, and multiple sclerosis are major neurological disorders that affect the CNS and present formidable challenges in the development of effective treatments 4-7. Neurodegenerative disorders (e.g., Alzheimer's and Parkinson's diseases) are characterized by abnormal protein accumulation in the brain and deterioration of cognitive and behavioral functions, resulting in cognitive impairment, neuronal death, and imbalanced glial cell activity, posing a major threat to the health and well-being of the older population 8, 9. Brain cancer is among the most aggressive and lethal primary intracranial tumors of the CNS and is known for its poor prognosis, elevated recurrence risk, and minimal survival rates 10.

The main challenge in treating CNS disorders is the presence of a blood-brain barrier (BBB), a highly selective and tightly regulated interface comprising endothelial cells, microglia, pericytes, astrocytes, and a basement membrane. Strong interactions at tight junctions bind these cells and limit transcellular transit 11. The BBB is essential for maintaining the CNS microenvironment because it separates circulating blood and brain interstitial fluids. It preserves brain homeostasis, facilitates communication between the central and peripheral systems, and regulates potential infections and immunological reactions 5, 12-14. However, the tight BBB structure presents a significant obstacle to the effective delivery of therapeutic agents, limiting the treatment of CNS disorders 8. During tumor progression, the integrity of the BBB is compromised, forming the blood-tumor barrier (BTB) 15. Although the BTB is more permeable than the normal BBB, its uneven permeability to both small and large molecules, along with irregular blood perfusion, often leads to insufficient drug accumulation within brain tumors. Consequently, the BBB and its pathological form, the BTB, remain the major limiting factors in achieving effective clinical treatment 12, 16. Patients with neurological disorders often require long-term drug administration, which can lead to side effects in non-target organs. Moreover, numerous drugs that are effective in treating these conditions lose their therapeutic potential because of the restrictive nature of the BBB. Consequently, treatment options for patients with neurodegenerative diseases and brain tumors remain limited 17, 18.

In recent decades, various invasive and noninvasive strategies have been developed to overcome the restrictive nature of the BBB and effectively target specific diseased areas within the brain 4, 19, 20. Researchers have attempted to design effective nanomedicines to overcome the obstacles in the treatment of CNS diseases. The clinical application of nanomedicine has opened new avenues for selective and targeted drug delivery systems. Nanoformulations, especially carbon dots (CDs), have become a significant center of attraction to treating major CNS disorders 21. The enhanced therapeutic efficacy of CDs is attributed to their high drug-loading capacity, long-term stability in biological fluids, biocompatibility, and ability for controlled drug release. Their ultrasmall size and unique surface chemistry enable them to effectively cross the BBB and deliver neurological drugs to the CNS 22-24. Table 1 compares CDs with established nanocarrier systems, such as liposomes, polymer nanoparticles, inorganic nanoparticles, and exosomes, in terms of their advantages, limitations, and features that may favor BBB transport, while also acknowledging current limitations and translational considerations.

**Figure 1:**
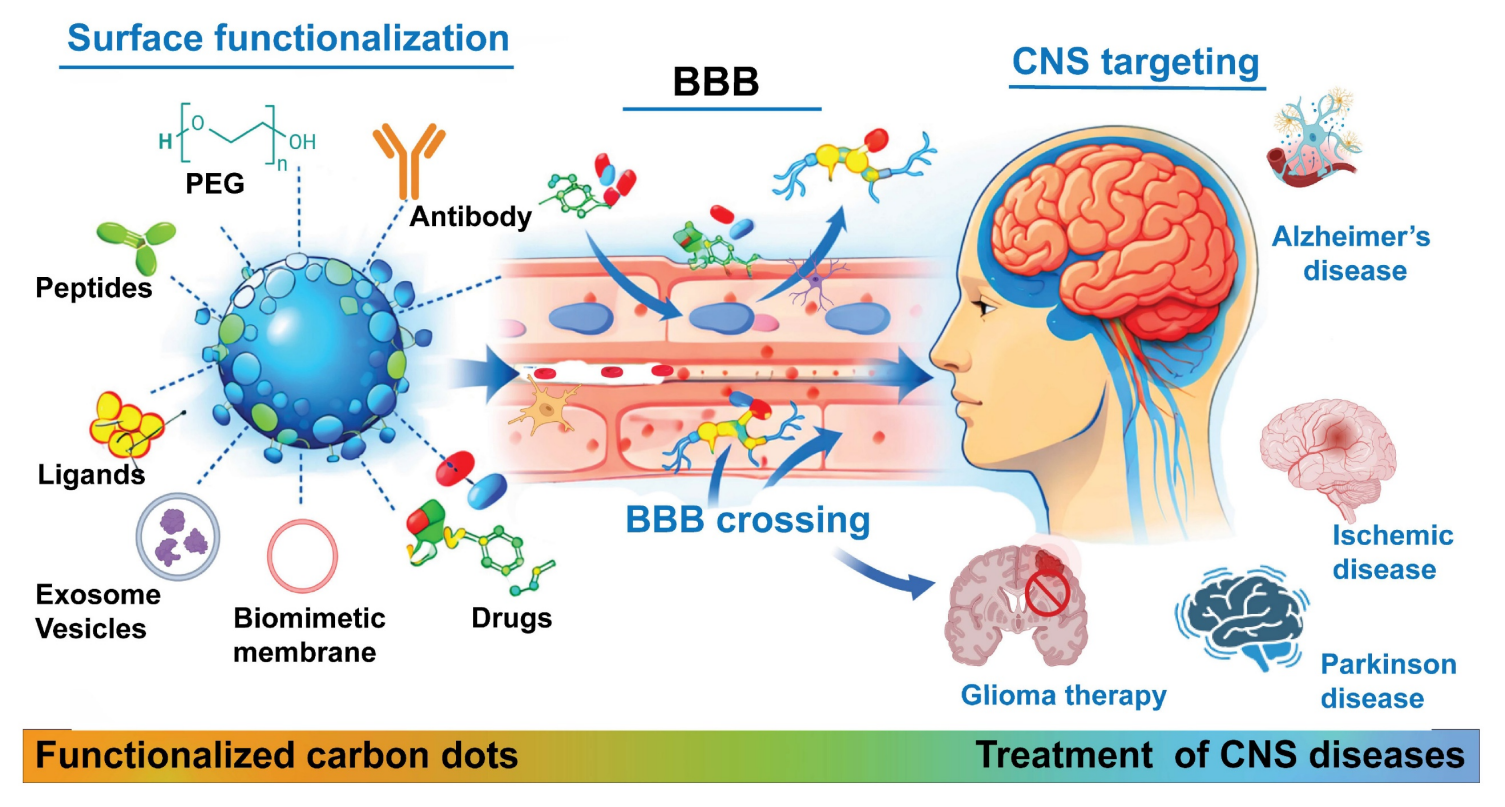
Schematic illustration of CD functionalization strategies to facilitate BBB penetration for imaging, therapy, or theranostic applications in CNS disease treatment. Created with BioRender.com.

In this review, we summarize the synthesis strategies, characterizations, and properties of CDs, discuss recent advances in the design of BBB-crossing CD-based nanomedicines for targeting CNS diseases, including surface modifications using targeting ligands, polymers, biomimetic membranes, and exosomes. We briefly discuss the modification strategies for CDs to facilitate BBB penetration, enabling targeted diagnosis and treatment of CNS diseases through modalities such as imaging, chemotherapy, gene therapy, antioxidant and anti-inflammatory treatments, photothermal therapy, photodynamic therapy, sonodynamic therapy and imaging-guided therapeutics (Figure 1). Finally, we highlight the current challenges and future perspectives of using CD-based nanomedicines for treating neurological disorders and brain cancer. This review offers a focused overview of recent advancements in CD-based nanomedicines for neurotheranostics.

## 2. Carbon dots

CDs were first discovered in 2004 by Xu *et al.* as a fluorescent nanomaterial formed as a byproduct during the purification of carbon nanotubes, where their unexpected fluorescence revealed a new class of zero-dimensional carbon-based nanomaterial 25. Numerous researchers have explored various strategies for CD synthesis and surface engineering, as well as their widespread applications 26-28. Early studies basically focused on their optical properties, positioning CDs as a cost-effective photostable alternative to traditional semiconductor quantum dots for imaging and sensing applications 29. Subsequent research advanced CD synthesis through particle-size control, surface functionalization, and heteroatom doping, improving quantum yield and emission tunability 27 and marking the transition of CDs from incidental fluorescent probes to rationally designed nanoplatforms. In recent years, CDs have been explored as multifunctional nanodrug delivery systems owing to their ultrasmall size, aqueous dispersibility, tunable photoluminescence, low toxicity, and emerging capability to cross biological barriers such as the BBB, expanding their role from traditional imaging agents to theranostic platforms 11, 23, 30, 31.

In this section, we introduce representative synthesis methods, characterization techniques, and optical properties of CDs relevant to surface functionalization. A detailed discussion of other aspects—such as fluorescence mechanisms, particle size, surface charge, and other properties—is beyond the scope of this review. Readers interested in a comprehensive coverage of these topics are referred to the existing review articles by Yue *et al.* 32, Ge *et al.* 33, Girum *et al.* 34, Alafeef *et al.* 35, Yan *et al.* 36, Sharma *et al.* 37 and related works.

### 2.1. Synthesis methods of CDs

**Figure 2:**
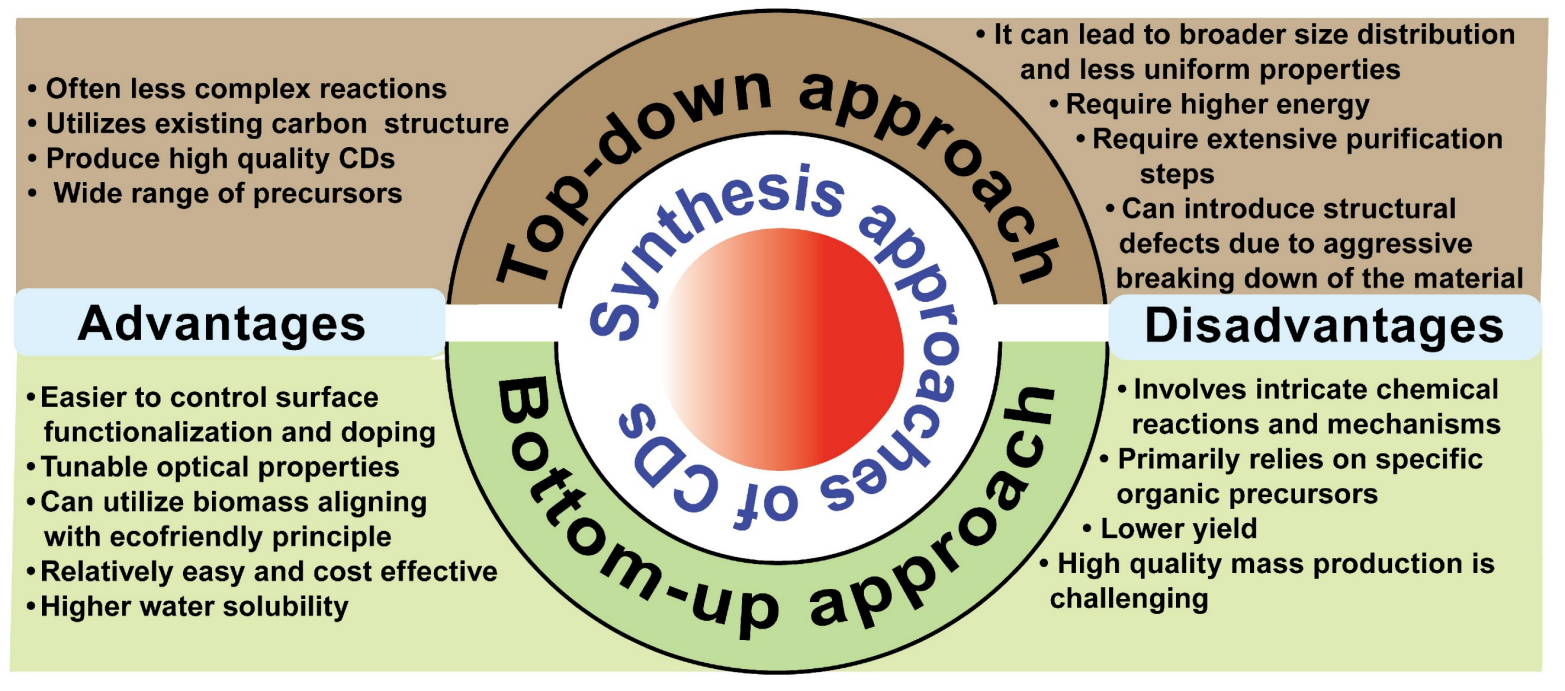
Advantages and disadvantages of top-down and bottom-up synthesis approaches of CDs.

Understanding the synthesis methods is important for tailoring CDs for specific biomedical applications. CDs can be synthesized through top-down or bottom-up approaches. Top-down approaches, including arc-discharge 25, plasma treatment 38, and laser ablation 39, break bulk carbon materials into small nano fragments, where the particle size and structure of CDs are controlled by adjusting physical parameters. However, its disadvantages include using harsh chemicals and physical conditions, producing uneven size distributions, irregular surface functionalities, and requirement for expensive equipment in some techniques 40. In contrast, bottom-up approaches, including pyrolysis microwave 30, hydrothermal 41, and ultrasonication 42, enable eco-friendly production, tunable composition, and tailored surface functional groups by the choice of precursors and reaction conditions. It still suffers from broad size distributions and batch-to-batch variability in fluorescence and quantum yield 40. We summarize the advantages and disadvantages of top-down and bottom-up methods in Figure 2.

In the synthesis of CDs, multiple interconnected parameters should be finely tuned to achieve a desirable size, photoluminescence, surface chemistry, and biocompatibility 43. Accordingly, the choice of synthesis method governs how these parameters are integrated and controlled during CD formation, as different approaches regulate precursor conversion, carbonization pathways, dopant incorporation, and surface passivation, ultimately determining the size distribution, quantum yield, and emission behavior of CDs 44. For example, hydrothermal, solvothermal, and pyrolysis methods determine reaction pathways and nucleation, which indirectly affect the optical properties and biological performances of CDs 45-47.

Dopant incorporation, solvent selection, temperature, and surface engineering are strongly governed by selection of synthesis strategies and together determine the structural and optical properties of CDs 48. For example, controlled incorporation of heteroatoms such as nitrogen, sulfur, or phosphorus during carbonization can modulate the electronic structures, defect states, and photoluminescence properties of CDs, enabling precise tuning of their overall properties 49-51. On the other hand, high boiling solvents commonly used in hydrothermal or solvothermal routes promote efficient dehydration, polymerization, and carbon core formations, ultimately leading to complete carbonization and enhanced optical properties 52. The thermal profile intrinsic to each specific synthesis method further regulates the degree of carbonization and passivation of surface groups, thereby determining particle uniformity and emission characteristics of the resulting CDs 49. In general, the surface groups on CDs, introduced either from precursors or via post-synthesis modification, govern their hydrophilicity, stability, and biofunctionalization capability. Therefore, the choice of synthesis technique serves as a central framework to tailor CDs for biomedical applications 33. Because numerous comprehensive reviews cover each synthesis approach in depth 33, 35, 40, 53, detailed synthesis approaches are not discussed here.

### 2.2. Characterization techniques of CDs

To evaluate the physical and chemical properties of CDs, various types of characterization techniques have been employed, including spectroscopic and microscopic methods 54. Prior to characterization, CDs must be purified via centrifugation, filtration, and dialysis to remove large aggregates, residual precursors, and small molecular fluorophores. The optical properties of CDs are analyzed using the clarified supernatant in quartz cuvettes to minimize scattering artifacts. Fluorescence properties of CDs are acquired from their excitation-emission spectra and three-dimensional photoluminescence mapping. Quantum yield of CDs is determined either using standards such as quinine sulphate or by absolute measurements employing a fluorescence spectrometer equipped with integrating sphere accessories 30, 55. Transit optical properties and excited state dynamics are evaluated using time-resolved approaches, including time-correlated single-photon counting, fluorescence upconversion, and transient absorption spectroscopy, to determine carrier lifetime and relaxation pathways 56. UV-visible absorption spectroscopy is routinely used to correlate the absorption properties of CDs with their emission behaviors.

Structural and morphological analyses of CDs are predominantly performed using high-resolution transmission electron microscopy (HR-TEM) and selected area electron diffraction, typically after depositing diluted CDs on an ultrathin grid 57. Atomic force microscopy (AFM) is used as a complementary approach to assess height profiles and dispersion properties of CDs 21. HR-TEM or AFM analysis generally indicates the particle size and size distribution of CDs in the range of 1-10 nm. Compositions and bonding environments are investigated using X-ray photoelectron spectroscopy (XPS), energy dispersive X-ray spectroscopy, Fourier transform infrared spectroscopy (FTIR), and Raman spectroscopy. Nuclear magnetic resonance, mass spectroscopy, and chromatographic techniques are additionally used to elucidate molecular composition and heterogeneity, particularly for CDs with molecular fluorophore-like characteristics 58, 59.

Despite extensive advanced analytical approaches, the characterizations of CDs still face challenges that require targeted improvements. One of the major challenges is the lack of standardized protocols for measuring key properties such as quantum yield, size, and lifetime, which leads to variability across studies and complicates direct comparison. Conventional methods for size distribution analysis, including TEM and AFM, often provide localized information and may not be accurate to reflect the hydrodynamic size states of CDs in biological media. Furthermore, characterization by FTIR and XPS is qualitative, which limits precise correlation between functional group density and optical features. Time-resolved spectroscopic investigations are not consistently used across studies, resulting in an incomplete understanding of charge-carrier dynamics and emission mechanisms. Future studies should focus on developing unified characterization standards, quantitative surface analysis methods, and *in situ* techniques to monitor dynamic structural and optical changes of CDs under physiological conditions. These efforts are essential for establishing structure-property relationships and enhancing the reproducibility required for downstream biomedical applications.

### 2.3. Properties of CDs

CDs are quasi-spherical nanomaterials featuring size-dependent and excitation-tunable photoluminescence, higher water solubility, excellent photostability, and abundant surface functional groups 60, 61. Their diverse properties make them suitable for a wide range of applications, such as sensing, bioimaging, drug delivery, photocatalysis, and optoelectronic devices 62. The structure of CDs generally consists of a carbon core and numerous functional groups on the surface, such as hydroxyl (-OH), carboxyl (-COOH), and amine (-NH_2_) groups 61. CDs are predominantly characterized by the coexistence of sp^2^ and sp^3^ hybridized carbon domains, which govern their fundamental physicochemical and optical properties 63. These intrinsic features underpin the use of CDs in biomedical fields, including bioimaging and bioanalysis. The sp^2^ hybridized carbon typically forms graphitic or polyaromatic domains that contribute to 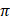-conjugation, electronic delocalization, and light absorption, while sp^3^ hybridized carbon constitutes amorphous regions and defect sites that are frequently decorated with oxygen- and nitrogen-containing functional groups 64, 65. Due to the presence of sp^2^ hybridized carbon, CDs are prone to π- π stacking, which can promote aggregation, quench fluorescence, and reduce stability 66. These interactions further affect protein corona formation and cellular uptake. Therefore, surface functionalization was commonly applied to maintain physicochemical and optical properties and balance electronic functionality with aggregation control in CDs design for biomedical applications.

Structural studies confirmed that most reported CDs consist of sp^2^-hybridized carbon domains embedded within an amorphous sp^3^-rich carbon framework functionalized with nitrogen- and oxygen-containing groups. X-ray diffraction studies reflect a high density of structural defects and partial disorder. These defects are related to functional moieties such as hydroxyl, carboxyl, and epoxy groups, playing critical roles in defining the electronic properties of CDs. Optical absorption and emission are governed by σ/n→σ* and n/π→π* transitions, where π-states originate from aromatic sp² carbon domains and n-states from lone pairs at structural defects 67. Variations in domain size and defect density modulate the effective energy gap, giving rise to size- and surface-dependent photoluminescence behaviors.

The optical properties of CDs are influenced by factors such as functionalization, particle aggregation, and solid concentrations 68. Heteroatom doping, such as nitrogen, can tune the optical transitions and affect the photoluminescence kinetics and lifetime of CDs 69, 70. The photoluminescence properties arise from a combination of quantum confinement in small sp^2^ domains, surface trap/defect states, and molecular fluorophore-like species 59. Surface engineering, particularly the introduction of oxygen-, nitrogen-, sulfur-, and phosphorus-containing functional groups, is also critical for tuning the properties of CDs for specific applications in sensing and biomedicine 71, 72. These functional groups profoundly influence the solubility, surface charge, bioconjugation capabilities, and optical behaviors of CDs 71. Oxygen-containing functional groups, such as -COOH, -OH, carbonyl (C=O), and ether (C-O-C) groups, are commonly found on the surface of CDs 71, 72. For instance, zirconium-doped CDs and pristine CDs exhibit prominent O-H, C=O, and C-O stretching vibrations in their infrared absorption spectra. These groups enhance the hydrophilicity of CDs, which is essential for maintaining stability and dispersibility in aqueous biological environments. Furthermore, these oxygen-containing functional groups provide reactive sites for subsequent chemical modifications, enabling the attachment of targeting ligands, therapeutic molecules, or biocompatible polymers for specific biomedical applications 71. In addition, surface defects and functional groups on CDs can generate tunable fluorescence across various wavelengths, including long-wavelength emissions that are advantageous for deep tissue bioimaging due to reduced scattering and absorption by biological tissues. The functionalization can also enhance the photostability of CDs, ensuring consistent performance in demanding biological environments 73, 74.

Rational design and precise control over oxygen-, nitrogen-, sulfur-, and phosphorus-containing functional groups through synthesis or post-functionalization are central to optimizing CDs for diverse biomedical applications. This surface engineering allows fine-tuning of solubility, charge, bioconjugation sites, and optical properties, ultimately advancing the development of highly effective CDs for sensing, diagnostics, and targeted therapeutic delivery.

## 3. Design of BBB-crossing CDs for CNS targeting

The BBB poses a significant obstacle to effective drug delivery to treat CNS disorders. Although the BBB remains intact in most neurodegenerative cases, it can undergo subtle disruptions that aggravate disease progression and restrict the delivery of therapeutic agents 75. Conversely, brain cancers frequently induce disrupted and uneven structures such as the BTB, which is more permeable than the normal BBB but heterogeneous. This irregular permeability and uneven blood flow hamper the uniform delivery of therapeutic agents 76. Intravenously administered nanoparticles accumulate in damaged brain tissue, despite their payload mechanisms. However, this inconsistent accumulation reduces the effectiveness of nanoparticle-based therapies for CNS disorders 77, 78. Therefore, the design of advanced drug delivery nanoplatforms capable of penetrating the BBB and targeting brain lesions is essential to effectively treat CNS disorders.

CDs are nanocarbon-based materials with excellent aqueous dispersibility, low cytotoxicity, and tailored surface properties 30, 79. Their tunable functional groups and high surface areas enable high drug loading, and their adjustable photoluminescence allows for drug tracking 80. Moreover, ultrasmall sizes (< 5 nm) can effectively cross the BBB between endothelial cells with 4-6 nm gaps 81, 82.

Various strategies have been adopted to effectively deliver CDs to the brain. For example, conjugation of CDs with peptides or ligands, such as angiopep-2 83, transferrin (TfR) 84, rabies virus glycoprotein 29 (RVG29) 85, TfR-binding peptide T7, or glucose analogs 86, can engage receptors such as low-density lipoprotein receptor-related protein 1 (LRP1) 87, TfR receptor, glucose transporter 1 (GLUT1), or L-type amino acid transporter 1 (LAT1) 88 on brain endothelial cells. These functionalization strategies facilitate receptor-mediated transcytosis (RMT) for the delivery of therapeutic CDs to the brain 89. Cationic CDs can be delivered to the brain via adsorption-mediated transcytosis (AMT) through electrostatic interactions with the negatively charged endothelial glycocalyx. However, this mechanism is less specific and increases off-target uptake 90. In pathological contexts, such as tumors or neuroinflammation, BBB/BTB distraction allows passive accumulation but with diminished targeting selectivity. Physical interventions, such as ultrasound, can temporarily open the BBB, allowing CDs to cross more effectively. Furthermore, intranasal injections provide a BBB-bypassing route, delivering CDs to the brain through the olfactory and trigeminal nerve pathways for efficient accumulation 91. However, intranasal delivery is influenced by mucociliary clearance and nasal residence time, as species-dependent anatomical differences between rodents and humans can affect clinical translation relevance. Brain accumulation following intranasal administration is typically assessed using fluorescence imaging, quantitative biodistributions, and histological analyses to confirm nose-to-brain transport 92, 93.

Research on CDs, specifically on BBB penetration, remains ongoing. CDs have been modified using various strategies to enhance their BBB penetration. This section outlines recent strategies for modifying CD surfaces to facilitate BBB penetration and target neurodegenerative diseases and brain cancer for effective diagnosis and treatment. Table 2 summarizes the general requirements for brain delivery, highlighting that the optimal design of CDs should be tailored to disease-specific alterations of the BBB and local pathophysiology. In neurodegenerative disorders such as Alzheimer's disease, the BBB largely exhibits reduced transporter efficiency, chronic inflammation, and impaired clearance mechanisms 94. In this context, ultrasmall CDs (less than 5 nm) with neural or mildly negative surface charge and targeting ligands are essential for RMT, while minimizing nonspecific immune activation. Additionally, antioxidants and anti-inflammatory CD formulations are relevant for mitigating oxidative stress and microglial overactivation associated with amyloid pathology 21, 95, 96.

In contrast, brain glioma presents highly heterogenous BBB regions that are partially disrupted, with abnormal vasculature and an immunosuppressive tumor microenvironment. For glioma treatment, CDs can be designed with targeting ligands such as epidermal growth factor receptor (EGFR) and TfR, as well as stimuli-responsive triggered drug release mechanism, to exploit the acidic and oxidative tumor milieu 97.

In ischemic stroke, BBB disruption is acute and transient, accompanied by elevated levels of reactive oxygen species (ROS) and inflammatory cytokines and increased vascular permeability. CDs formulated for treating this condition prioritize rapid BBB crossing, ROS scavenging capacity, and short systemic retention to reduce secondary injury. More specifically, biodegradable CDs with strong ROS scavenging and anti-inflammatory activity are suited for this setting, supporting neuroprotection and functional recovery while ensuring efficient renal clearance 98.

Overall, disease-specific considerations underscore that the design of BBB-penetrating CDs should not follow a universally applicable approach but should be tailored to the dynamic and pathological features of each neurological condition. Explicitly aligning CD physicochemical properties with disease-specific BBB alterations is essential for improving therapeutic efficiency and accelerating clinical translation.

### 3.1. Surface modification with a targeting ligand

**Figure 3:**
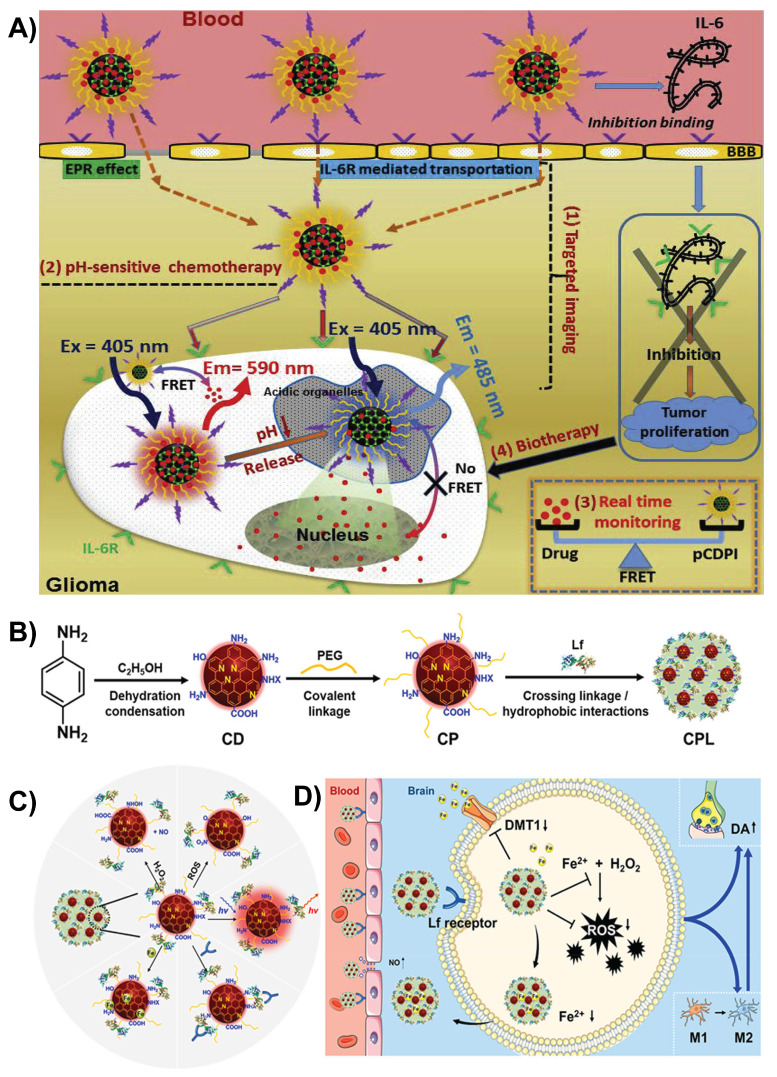
A) Schematic illustration of the *in vivo* delivery of multifunctional CDs for targeted imaging, pH-responsive chemotherapy, real-time monitoring, and biotherapy. Reproduced with permission 99 Copyright 2017, Elsevier. B) Schematic illustration of the synthesis of different nanoformulations. C) Multiple functions of Lf-modified CDs. D) Illustration of iron ion-sequestering and antioxidative CD-based nano formulation with NO release for Parkinson's disease treatment. Reproduced with permission from 103, copyright 2024, Elsevier.

By modifying CDs with peptides or ligands, their ability to connect with receptors on endothelial cells can be enhanced. The addition of functional groups, such as -COOH, -OH, -C=O, or imine (-C=N), facilitates their passage across the BBB through receptor-mediated transport, allowing for better targeting of disease-related lesions 81, 90. Wang *et al.* developed CDs linked with interleukin-6 (IL-6) peptide fragments and doxorubicin (DOX) to target IL-6 receptors for targeted drug delivery. This dual-targeting approach helps the CDs cross the BBB efficiently via RMT and reach deep into the glioma tissues. These smart CDs remained stable at the normal body pH but released DOX in the acidic tumor environment, making drug delivery more precise (Figure 3A). The fluorescence of CDs also allows real-time tracking of the drug location. In animal studies, these CDs could effectively cross the BBB, target gliomas, and release drugs in a controlled manner, paving the way for potential imaging-guided brain cancer treatments 99.

Recent advances have shown that CDs modified with ligands/peptides demonstrate substantially improved ability to penetrate the BBB via RMT. For example, TfR-conjugated CDs (TfR-CDs) selectively bind to TfR receptors on endothelial cells, allowing uptake in zebrafish models, whereas unmodified CDs do not cross the BBB 84. Similarly, glucose-derived CDs (GluCDs) with inherent -OH and -C=O functional groups use GLUT1-mediated transport to penetrate the BBB in zebrafish and rat models, allowing the delivery of fluorescent payloads without additional ligand conjugation 86. Recently, Chang *et al.* developed curcumin-derived CDs (Cur-CDs) using an eco-friendly one-step dry-heating method to enhance the delivery of neuroprotective agents across the BBB for Alzheimer's disease treatment. By retaining curcumin's phenolic -OH and β-diketone groups, Cur-CDs offer high solubility, biocompatibility, and improved BBB permeability due to their small size and functionalized surfaces. In SH-SY5Y cells, these CDs were nontoxic and effectively inhibited Aβ fibrillization and tau hyperphosphorylation, which are key pathologies of Alzheimer's disease. This study highlights surface-functionalized CDs derived from natural compounds as a scalable platform for crossing the BBB and targeting multiple pathways involved in Alzheimer's disease 100. Furthermore, this work exemplifies that rational selection of precursors and synthesis approaches can transform CDs from fluorescent materials to disease-specific biological platforms. Incorporating such disease-informed synthesis strategies will be essential for bridging the gap between laboratory-scale CD development and meaningful clinical translation.

### 3.2. Polymer coating strategy

Coating CDs with biocompatible polymers, such as polyethylene glycol (PEG) or chitosan, can make them more stable, less toxic, and better at crossing the BBB through both active and passive mechanisms 101. For example, CDs coated with chitosan or albumin can undergo AMT to cross the BBB. However, efflux pumps in the brain may reduce the retention time of these CDs in the brain 102.

Huang *et al.* designed red-emissive CDs by modifying them with PEG-lactoferrin (PEG-Lf) to improve brain delivery of the drug to treat Parkinson's disease. This modification allows CDs to cross the BBB in two ways: by temporarily opening the barrier with nitric oxide (NO) and via lactoferrin RMT (Figure 3B and C). Once in the brain, CDs exert multifaceted neuroprotective effects by neutralizing harmful ROS owing to their antioxidant properties, release NO to reduce neuroinflammation, and bind excess iron to prevent oxidative stress via the Fenton-like reaction. These PEG-Lf-modified CDs also supported real-time imaging and showed promise in mouse models of Parkinson's disease by improving motor deficits and reducing neurodegeneration (Figure 3D). The PEG-Lf coating modulated the surface charge of the CDs to be more neutral, which minimized unwanted interactions, boosted the uptake by lactoferrin receptors, and lowered toxicity 103.

Apart from promoting BBB penetration, this strategy improves the circulation time, stability, biocompatibility, and targeting capability of CDs to treat CNS diseases. The most common polymers used to coat CDs for crossing the BBB are PEG, chitosan, polyethyleneimine (PEI), polysorbate 80 (Tween 80), and poly(lactic-co-glycolic acid). These coatings enhance hydrophilicity, help evade immune clearance, provide mucoadhesion, promote AMT and RMT, and enable controlled drug release.

### 3.3. Modification with biomimetic membranes and exosomes

CDs can be enveloped in membranes sourced from cells (such as macrophages or mesenchymal stem cells) to replicate biological structures, improving their capacity to penetrate the BBB via cell-mediated transcytosis or immune cell infiltration 104. Xie *et al.* developed a biomimetic nanoplatform for the targeted treatment of Alzheimer's disease. Nitrogen-doped CDs (N-CDs) are enclosed within macrophage cell membranes, allowing them to cross the BBB and specifically target Aβ plaques. The N-CDs perform dual roles in regulating metal ion homeostasis to disrupt toxic metal Aβ interactions and delivering photothermal therapy via near infrared (NIR) irradiation to disaggregate Aβ plaques. This approach enhances therapeutic precision, reduces off-target effects, and shows promise in targeting Aβ plaques and modulating the Alzheimer's disease microenvironment 105. While the macrophage membrane camouflage provides targeting and BBB crossing, potential immunogenicity, long-term biosafety, and large-scale production of the nanoplatform remain to be established in future preclinical studies.

**Figure 4:**
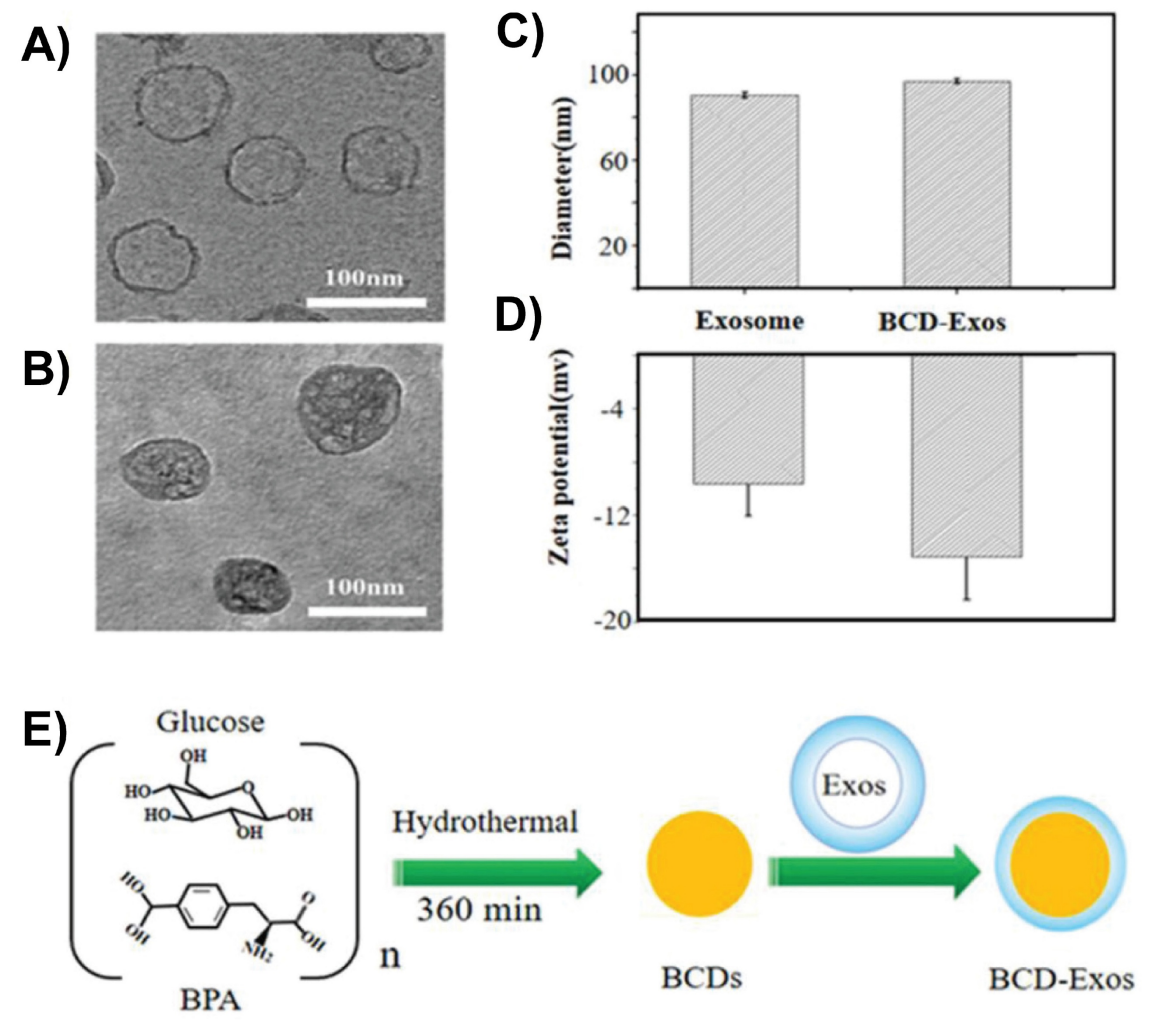
TEM images of A) Exosomes and B) BCD-Exosomes. C) Hydrodynamic size distributions and D) zeta potentials of BCDs and BCD-Exosomes in PBS. E) BCD-Exosome preparation. Reproduced with permission from 106, copyright 2021, Wiley-VCH.

Exosomes are natural nanocarriers with low immunogenicity and inherent BBB-crossing capabilities. CDs can be incorporated into exosomes to deliver drugs and imaging agents to the brain. Boron-enriched CDs (^10^BCDs) were encapsulated within macrophage-derived exosomes to create a biomimetic nanoplatform (BCD-exosome) for targeted glioma therapy using boron neutron capture therapy (BNCT). Exosome coating considerably enhanced the ability of CDs to cross the BBB by leveraging the natural targeting and transcytosis mechanisms of macrophage vesicles. As shown in Figure 4A-D, TEM and dynamic light scattering analysis confirmed successful encapsulation of BCDs in exosomes, yielding a size of approximately 100 nm. Upon neutron irradiation, BCD-exosomes (Figure 4E) delivered a localized dose (~8.4 Gy), resulting in complete tumor ablation and 100% survival of the treated mice 106.

**Figure 5:**
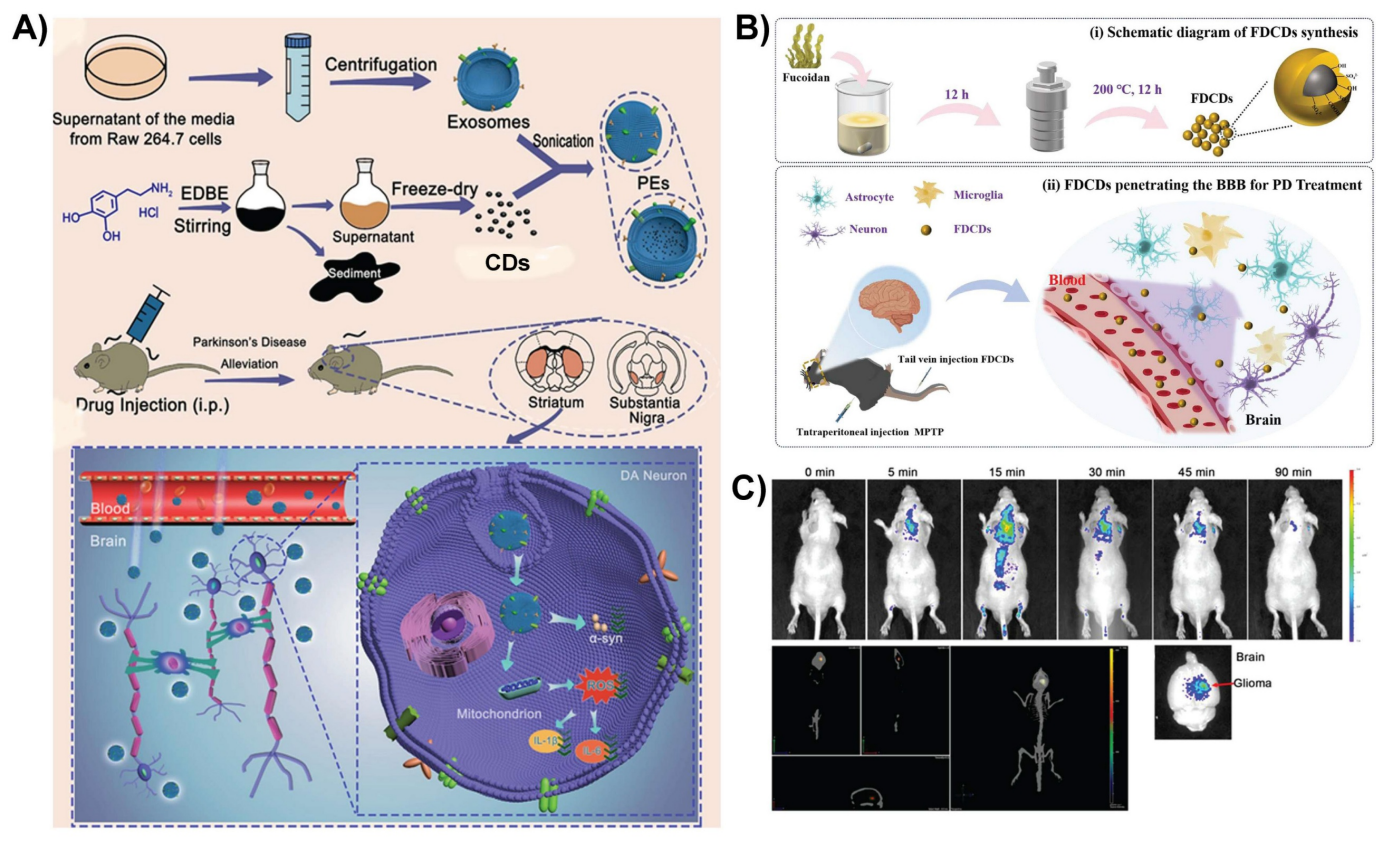
A) Synthesis and modification of PCDs@Exosomes, along with the proposed mechanism for treating Parkinson's disease. Reproduced with permission. 107 Copyright 2025, Elsevier. B) Schematic illustration of fucoidan-derived CDs crossing the BBB for Parkinson's disease treatment. Reproduced with permission from 21, copyright 2025, Elsevier. C) *In vivo* and* ex vivo* imaging of glioma-bearing mice following tail vein injection of CD-Asp; whole-body distribution over time, 3D reconstruction of brain localization at 20 min post-injection, and *ex vivo* brain imaging at 90 min post-injection. Reproduced with permission from 108, copyright 2015, American Chemical Society.

Similarly, Yang *et al.* reported macrophage-derived exosome-coated polydopamine-CDs (PCDs) of 2-4 nm in size for formulating a biomimetic nanoplatform (PCDs@Exosomes) for Parkinson's disease therapy. Exosomes facilitate the BBB-crossing ability of CDs by exploiting the uptake mechanisms of macrophage extracellular vesicles and their homing nature to target inflamed neural tissues (Figure 5A). After crossing the BBB, PCDs effectively mitigate both motor and non-motor symptoms in Parkinson's disease mouse models by exerting antioxidant and anti-inflammatory effects, decreasing α-synuclein aggregation, and promoting neuronal viability in areas such as the substantia nigra, striatum, and prefrontal cortex 107. In general, modifying CDs with biomimetic membranes or exosomes can enhance BBB penetration by utilizing cell-mediated transport for targeted homing. These modifications also promote immune evasion, prolong circulation time, and improve biocompatibility.

### 3.4. Other modification strategies for CDs

In addition to surface modification in CDs, other strategies have been developed to improve the capacity of CD-based nanomedicines to traverse the BBB and treat neurodegenerative disorders. Fucoidan-derived CDs penetrate the BBB via various complementary pathways. Due to their extremely small size (less than 4-6 nm), they can passively diffuse through nanometer-sized gaps between endothelial cells via paracellular transport. This mechanism works well when the BBB is compromised, which is frequently observed in neurodegenerative conditions, such as Parkinson's disease, where inflammation and oxidative stress increase BBB permeability (Figure 5B). Furthermore, the negative charge on the CD surface, resulting from the sulfate groups of fucoidan, induces electrostatic interactions with positively charged sites on the BBB or carrier proteins, thus promoting AMT and improving penetration. The combination of their ultrasmall size, surface charge, and altered state of BTB supports the effective delivery of fucoidan-derived CDs into the brain, establishing them as promising options for the targeted treatment of Parkinson's disease and other CNS disorders 21. Zhang *et al.* found that CDs made from D-glucose and L-aspartic acid (CD-Asp) can cross the BBB and accumulate in glioma tissues without external targeting agents. The small size of these CDs allows them to passively cross the BBB. Their surface chemistry, which includes -NH_2_ and -COOH groups from L-aspartic acid, may increase glioma cell uptake. *In vivo* fluorescence imaging demonstrated that 15 min after intravenous injection, CD-Asp showed a substantially greater signal in glioma than in normal brain regions (Figure 5C). Control CDs containing D-glucose or L-glutamic acid alone showed negligible brain targeting, underlining the unique self-targeting capabilities of CD-Asp for noninvasive glioma diagnosis 108.

A targeted theranostic platform for Alzheimer's disease was developed by conjugating CDs to triple-functionalized human serum albumin (HSA) to create a multifunctional nanocomposite. Three ligands known to promote transport across the BBB—angiopep-2 (a BBB-penetrating ligand), neurotropic peptide (RVG29), and TfR (a TfR receptor ligand)—were added to the HSA. These surface changes facilitate RMT and allow CDs to pass through the BBB and accumulate in the brain. After entering, the CDs offer fluorescent imaging capabilities and collaborate with HSA to scavenge ROS, bind metal ions, and prevent Aβ aggregation. This multitarget strategy is suitable for simultaneous diagnosis and treatment of Alzheimer's disease 23.

### 3.5. Mechanistic insights into CD functionalization strategies for disease-specific CNS pathology

Table 2 summarizes representative functionalization strategies of CDs and their relative efficiency, selectivity, and translational limitations. However, more explicit comparisons of existing strategies are warranted in the context of disease-specific pathology. Ligand-mediated functionalization strategies offer higher targeting specificity via RMT in multiple preclinical models. However, this method is constrained by receptor saturation, competitive binding with endogenous ligands, and disease-dependent variability in receptor expression in neurodegenerative conditions 99. These limitations highlight the need to align ligand selection and density with the pathological state of the BBB. In contrast, polymer-based surface modification could enhance circulation stability and reduce nonspecific protein adsorption rather than conferring active targeting. Despite their improved pharmacokinetics and reduced immune clearance, the BBB-crossing efficacy of polymer-coated CDs is generally lower than that of ligand-functionalized CDs and relies largely on passive diffusion or nonspecific endocytosis. Moreover, excessive polymer shielding may hinder cellular uptake and intercellular trafficking 81. Such approaches may therefore be suitable for conditions involving transient BBB disruption, such as ischemic stroke, rather than diseases with intact BBB.

Biomimetic functionalization approaches provide a multifactorial mechanism for BBB penetration by combining immune evasion, prolonged circulation, and endogenous receptor engagement. These approaches display superior biological mimicry and enhanced accumulation in brain tissue 107. However, they face significant challenges related to batch-to-batch variation, complex preparation, and limited scalability, which hinder their clinical translation 109.

Importantly, the effectiveness of the above functionalization strategies is closely related to upstream synthesis preference, including precursors and reaction parameters, which can determine the overall physicochemical properties of CDs. The lack of connection among synthesis approaches, functionalization mechanisms, and disease-specific pathology could be a major bottleneck for the clinical translation of CDs. No single functionalization strategy is universally optimal. Future CD design should prioritize hybrid, or modular functionalization approaches that balance targeting precision, biological stability, and manufacturability, while being guided by the pathological features of specific CNS diseases.

## 4. Diagnosis and treatment of brain diseases using BBB-penetrating CDs

**Figure 6:**
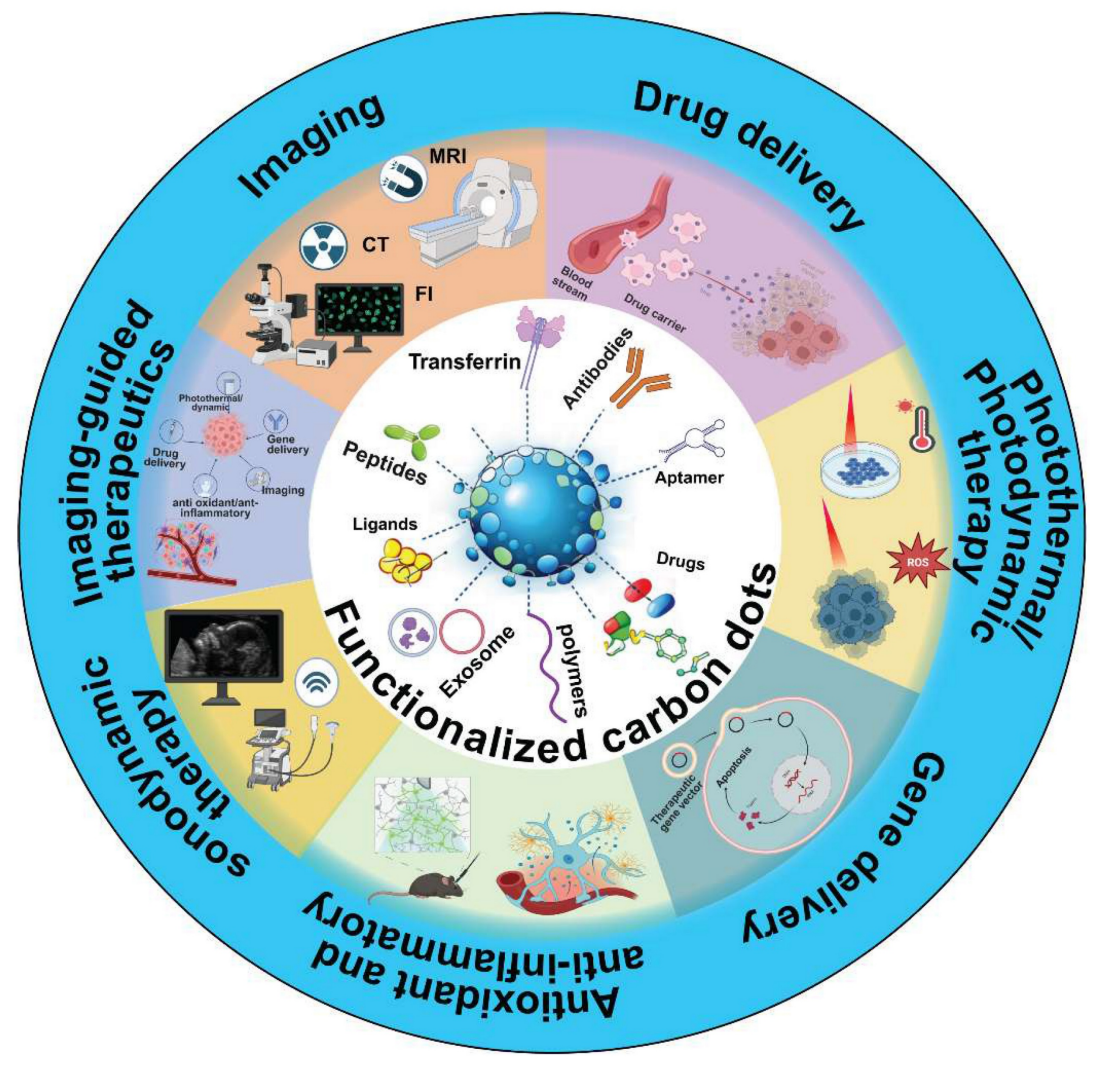
Schematic overview of functionalized CDs capable of crossing the BBB for theranostic applications in CNS diseases. Created with BioRender.com.

Building upon the above CD design strategies to enable BBB penetration, various nanomedicine formulations have been developed for imaging, treatment, and theranostic management of brain disorders. In this section, we summarize the key advancements in CD-based nanomedicines, focusing on their applications in imaging, drug delivery, gene delivery, photothermal therapy, photodynamic therapy, sonodynamic therapy, antioxidant and anti-inflammatory treatment, and theranostic approaches for CNS diseases (Figure 6). A comprehensive overview of the mechanism of action of CDs in different treatments and diagnostic strategies for neurodegenerative diseases and brain cancer is presented in Table 3. Although some of these applications were briefly mentioned in the previous sections, a more detailed examination of their roles and potential in the management of CNS diseases is presented below.

### 4.1. Imaging

**Figure 7:**
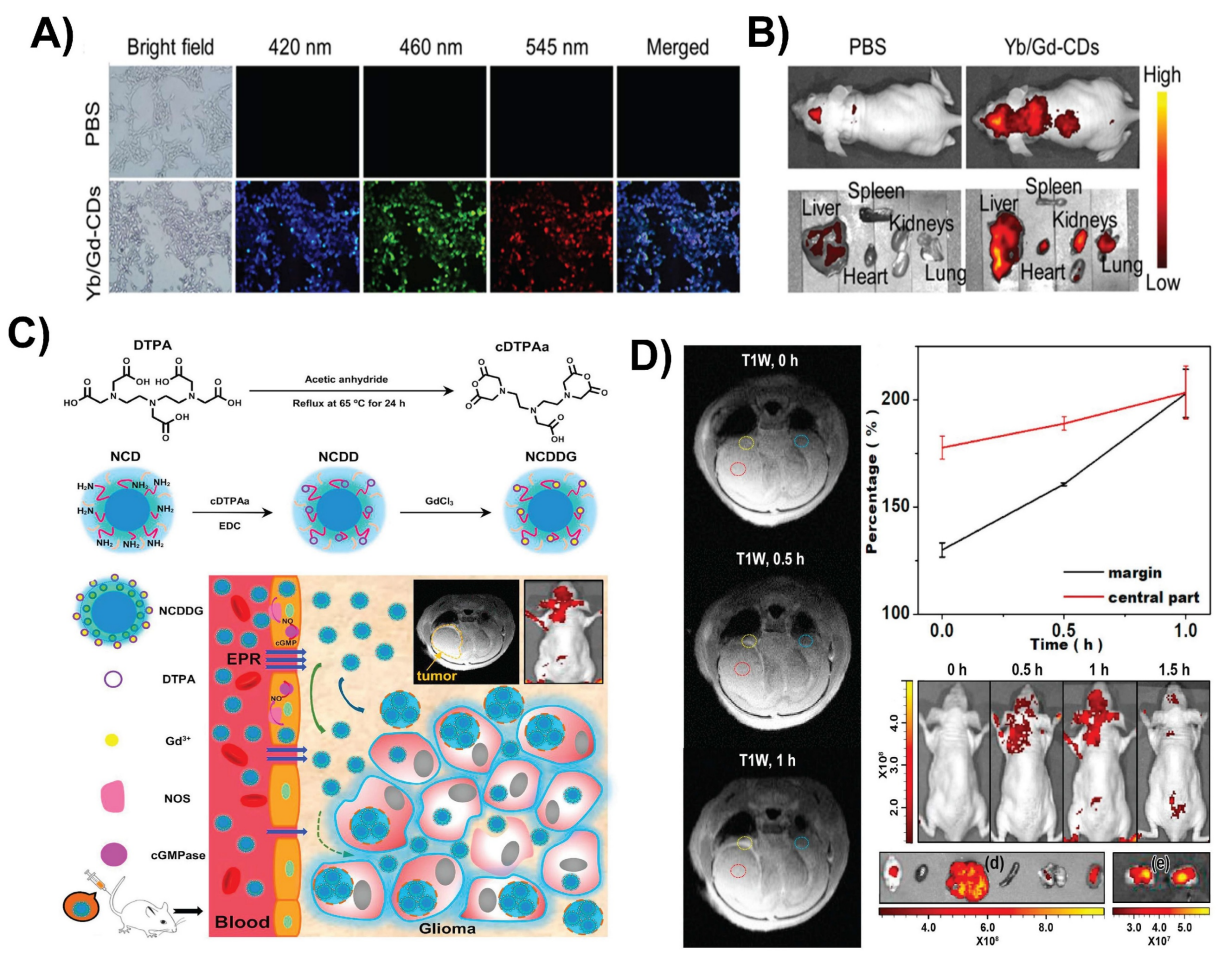
A) Confocal fluorescent microscopic images of 4T1 cells incubated with PBS and Yb/Gd-CDs. B) In vivo fluorescence images nude mice and major organs (heart, liver, spleen, lungs, and kidneys) after intravenous injection of PBS and Yb/Gd-CDs (excitation: 430 nm; emission: 500 nm). Reproduced with permission from 57, copyright 2024, Elsevier. C) Schematic diagram of the preparation of NCD coupled with clinical Gd-DTPA and its major biological effects. D) *T_1_*-MR images of a representative nude mouse pre-injected with NCDDG (left panel); *T_1_*-MR signal-to-background ratio for tumor margin and center (upper right); fluorescence images of the mouse at various time points (middle right); fluorescence images of major organs (brain, heart, liver, spleen, lung, and kidney) and two cross-sections of the brain. Reproduced with permission from 113, copyright 2018, Elsevier.

CDs are nontoxic and display excellent photoluminescence and transverse BBB permeability, making them promising candidates for noninvasive imaging of the brain. Surface functionalization, doping, and biomimetic coating can enhance their imaging performance 110. Manganese-doped CDs (Mn-CDs) were synthesized via a one-pot microwave-assisted method, resulting in ultrasmall particles exhibiting excitation-dependent fluorescence and exceptionally high *T₁* magnetic resonance imaging (MRI) relaxivity. Following systemic administration in a mouse glioma model, Mn-CDs enabled *in vivo T₁-*weighted MRI to distinctly enhance the contrast within the glioma region and reveal tumor margins. *Ex vivo* fluorescence imaging of resected brain tissue confirmed the selective accumulation of Mn-CDs in brain tumors, as evidenced by localized bright photoluminescence. Owing to their low cytotoxicity, strong MRI contrast, and effective optical imaging capabilities, Mn-CDs have strong potential as dual-modal nanoprobes for the precise detection and intraoperative localization of small brain gliomas 111. Gadolinium-ytterbium co-doped CDs (Yb/Gd-CDs) were created via a one-pot hydrothermal method with an ultrasmall size, excellent colloidal stability, strong fluorescence, and robust computed tomography (CT) and MRI contrast. *In vivo* imaging in mice demonstrated bright fluorescence signals (Figure 7A and B), clear CT visualization of tissues at high X-ray attenuation (~40 HU/g/L), and *T₁-*weighted MRI with a T₁ relaxivity of ~11.16 mM⁻¹ s⁻¹. This multimodal platform supports precise anatomical localization (MRI/CT) along with sensitive optical tracking, which is ideal for detecting brain tumors, such as gliomas 57. In addition, Gd-doped CDs (~2-8 nm) derived from starch showed high MRI relaxivity and stable red emission.

In cellular models, they are localized within the cytoplasm and produce robust contrast enhancement, highlighting their potential for brain mapping and imaging gliomas. These CDs also exhibit bright and stable fluorescence, ultrasmall size (<10 nm), and optimized surface characteristics, enabling them to cross the BBB and selectively target brain tumors. Their high MRI relaxivity enhances the imaging contrast, whereas real-time optical imaging provides dynamic visualization. These features support multimodal imaging capabilities (fluorescence imaging + MRI + CT), improve detection accuracy, and allow cross-validation across different imaging modalities 112. Similarly, polymer-coated N-CDs covalently linked with clinical-grade Gd-DTPA formed a dual-modal nanoprobe for MRI and fluorescence imaging of gliomas (Figure 7C). The polymer shell enhances biocompatibility, whereas its ultrasmall size (~5-10 nm) facilitates BBB penetration and tumor targeting via the enhanced permeability and retention effect. Compared with free Gd-DTPA, Gd-CDs exhibit higher T₁ relaxivity and stronger fluorescence. As shown in Figure 7D, glioma-bearing mice displayed a clear, time-dependent MRI signal enhancement, with fluorescence imaging of brain sections confirming precise tumor localization 113. This platform offers high-resolution, biocompatible glioma imaging with strong MRI and optical contrast.

### 4.2. Drug delivery

**Figure 8:**
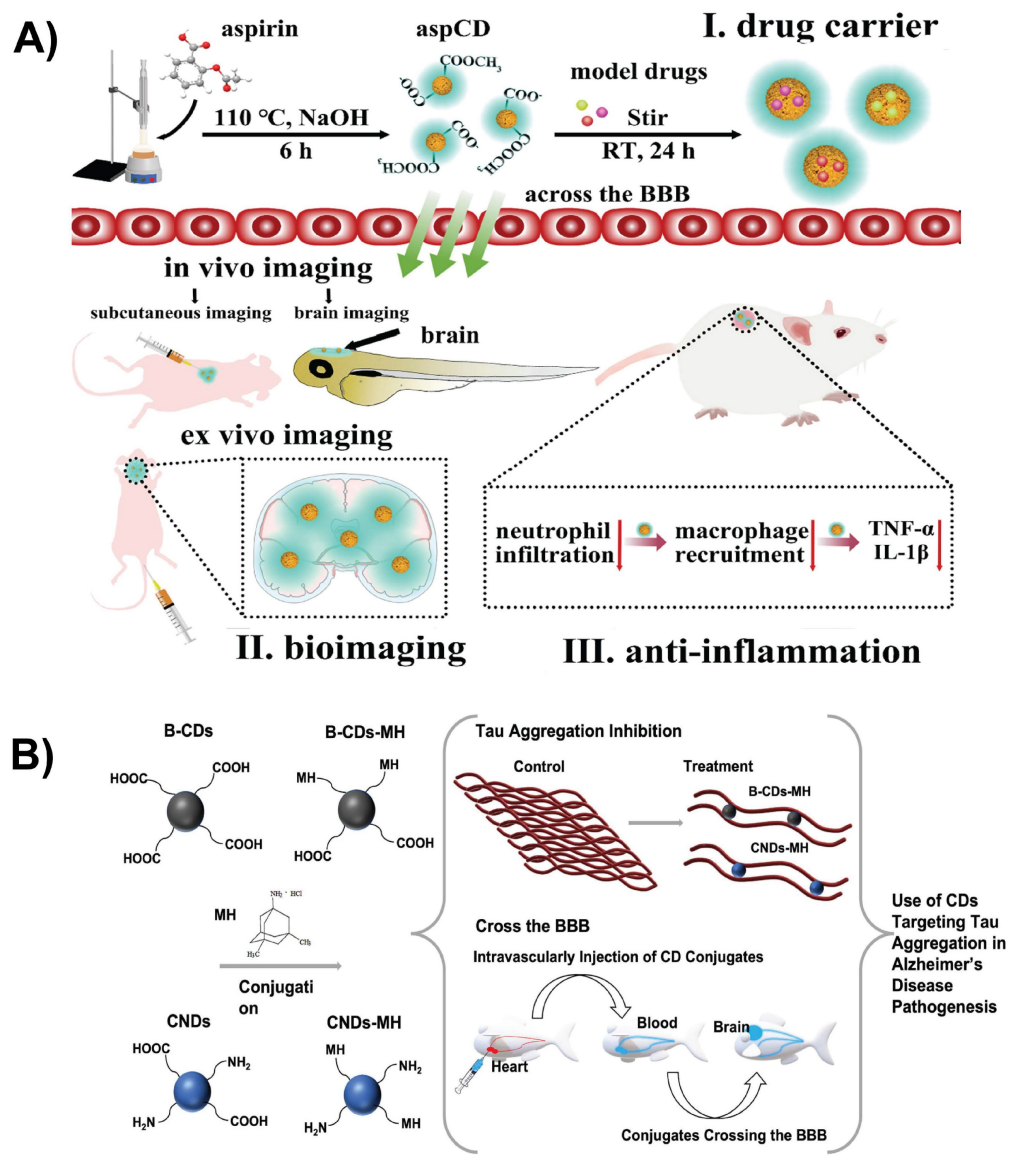
A) Schematic representative of the synthesis and application of aspirin-derived CDs and their applications in drug loading, bioimaging, and anti-inflammation. Reproduced with permission from 117, copyright 2021, Elsevier. B) Schematic representation of CDs conjugated with memantine and their applications in delivering memantine to treat Alzheimer's disease. Reproduced with permission from 118, copyright 2022, Elsevier.

Drug delivery systems are designed to transport drugs to specific sites in the body for effective drug interaction. Nanostructured materials improve drug delivery by enhancing the absorption, distribution, and elimination of drugs. Nanotechnology enables targeted delivery, improved solubility of poorly water-soluble drugs, co-delivery of multiple therapies, transport of large macromolecules, and real-time monitoring using imaging agents 114. Triple-conjugated CDs functionalized with TfR and loaded with two chemotherapeutic drugs, epirubicin and temozolomide, were designed for targeted brain delivery. The TfR on CDs facilitates RMT across the BBB, enhancing their accumulation in glioblastoma cells. This dual-drug delivery system induces synergistic chemotherapeutic effects at lower doses, improving tumor inhibition while minimizing the off-target toxicity. The small size and surface modifications of CDs promote cellular uptake and controlled drug release within the tumor microenvironment, making them promising nanocarriers to effectively treat brain cancer 115. TfR is highly expressed in the BBB and in many brain tumors; therefore, many nanoplatforms engage TfR-mediated endocytosis for cellular uptake. Li *et al.* formulated a nanocarrier in which CDs are covalently conjugated with the targeting ligand TfR and the drug DOX for brain tumor treatment. The conjugated CDs showed a more significant uptake in pediatric brain tumor cell lines compared to the free DOX because the TfR moiety facilitated receptor recognition and internalization 116. In another study, the small size and surface chemical properties of CDs promoted BBB penetration in both zebrafish and mouse models. CDs fabricated by hydrothermal carbonizations of aspirin exhibited a stable photoluminescence for imaging while maintaining good biosafety both *in vitro* and *in vivo* (Figure 8A). In addition to imaging, it functioned as an anti-inflammatory agent with the ability to load and deliver drugs of varying polarities 117. Zhang *et al.* employed CDs as a carrier to deliver the Alzheimer's drug memantine across the BBB *in vivo*. They synthesized carbon nitride CDs and black CDs functionalized with memantine using N-ethyl-N′-(3-dimethylaminopropyl)carbodiimide/N-hydroxysuccinimide amidation (Figure 8B). Due to their diverse surface functionalities and small size, these conjugates facilitated efficient transport across the BBB in the zebrafish model. After penetrating the BBB, CDs not only delivered memantine but also inhibited tau protein aggregation. These findings indicate that CDs serve as nanocarriers for drug delivery across the BBB and as functional inhibitors of disease-related biomolecular processes in the brain 118.

### 4.3. Gene delivery

**Figure 9:**
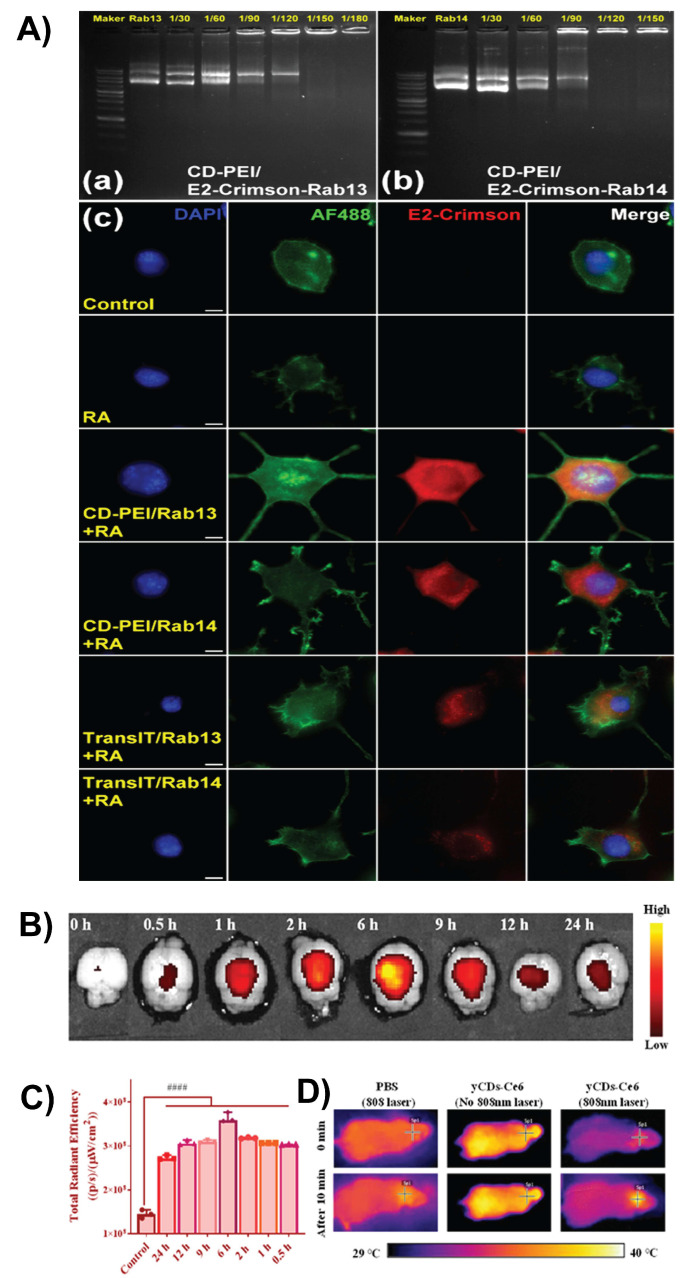
A) Agarose gel analysis of CD-PEI/plasmid complexes for (a) E2-Crimson-Rab13-Q67L and (b) E2-Crimson-Rab14 at DNA:CD-PEI ratios of 1/30-1/180. (c) Fluorescence images of N2a cells after 4 days showing expression of Rab13-Q67L or Rab14 delivered by TransIT (positive control) or CD-PEI. E2-Crimson (red), nuclei (DAPI, blue), and F-actin (Alexa Fluor 488, green). Scale bar: 10 μm. Reproduced with permission. 120 Copyright 2023, American Chemical Society. B) Fluorescence signals observed in the brain tissues of mice 24 h after tail vein injection of yCDs-Ce6 and C) Corresponding fluorescence signals of yCDs-Ce6. D) Thermal graphs of mice brain tissues treated with 808 nm laser irradiation for 10 min after intravenous injection of PBS and yCDs-Ce6. Reproduced with permission from 124, copyright 2022, American Chemical Society.

Gene delivery requires transporting genetic material (such as DNA, siRNA, or mRNA) across the BBB. This strategy aims to correct defective genes, silence harmful genes, and promote the expression of therapeutic proteins. It holds promise for treating conditions such as gliomas, Parkinson's disease, and Alzheimer's disease 17, 102, 119. CDs offer a safe and effective nonviral platform for gene delivery. Functionalized CDs, especially those with amines or glucose, can bind to genetic materials and cross the BBB with minimal toxicity. Their small size, tunable surface, and intrinsic fluorescence also allow tracking of delivery and therapeutic effects 86. Zhang *et al.* prepared ultrasmall, fluorescent, and positively charged pentaethylenehexamine-modified CDs (PCDs) using a facile microwave method and noncovalently bound them to plasmid DNA at a 2:1 mass ratio, achieving high transfection efficiency and notably lower cytotoxicity than conventional polyethylenimine (PEI)-based vectors. Crucially, these PCDs crossed the BBB in zebrafish models, demonstrating potential for non-viral gene delivery and bioimaging in CNS applications, such as neurodegenerative disease treatment and glioma therapy 119. Researchers synthesized CDs using nitric acid reflux and subsequently coated them with PEI. The modified CDs possess a positive charge and strong fluorescence and serve as both imaging probes and gene-delivery vehicles. Gel retardation analysis suggests that these CDs successfully bind to Rab13-Q67L and Rab14 plasmids (Figure 9A) and are readily internalized by neurons. Fluorescence images of N2a cells demonstrate cellular uptake and strong expression of Rab13-Q67L and Rab14 (E2-crimson, red), with minimal background in controls (Figure 9A). These results are consistent with the reported high transfection efficiency of up to 97%, while exhibiting minimal cytotoxicity. Following delivery, CDs significantly enhanced the expression of Rab13 and Rab14, leading to notable neurite outgrowth, with over 56% of neurons displaying neurites longer than twice the diameter of their cell bodies. This demonstrates the potent capacity of CDs to promote neurite sprouting and elongation. This study presents a highly effective fluorescent nanocarrier system for non-viral gene delivery to the nervous system 120.

### 4.4. Photothermal and photodynamic therapy

Photothermal and photodynamic therapies are light-activated treatment strategies that can be enhanced using CDs for brain diseases, such as glioma. In photothermal therapy, CDs with strong NIR absorption convert light into localized heat, inducing hyperthermia and targeted tumor cell death 121. In photodynamic therapy, CDs act as or carry photosensitizers (e.g., Ce6 or porphyrin), which, upon red or NIR light activation, trigger apoptosis 122. CDs can effectively cross the BBB, accumulate in brain lesions, and offer dual functionality for both therapy and fluorescence imaging, enabling precise image-guided treatment with minimal damage to the surrounding healthy tissue. Recently, Fan *et al.* developed CDs with dual photodynamic and photothermal therapy capabilities for brain applications. These CDs are engineered to cross the BBB, allowing targeted treatment of Aβ plaques associated with Alzheimer's disease. Under laser irradiation, the CDs generate ROS for photodynamic therapy-mediated oxidative degradation of Aβ plaques, while also producing localized heat for photothermal therapy, enhancing plaque disruption and clearance. Their multifunctional properties, which combine BBB permeability, ROS generation, and thermal effects, make them promising noninvasive therapeutic tools for targeting neurodegenerative pathologies through combined photothermal/photodynamic therapy mechanisms 123. Yan *et al.* reported a multifunctional nanoplatform composed of CDs and a photosensitizer (Ce6), forming nanoassemblies (CD-Ce6) with anti-amyloid, antimicrobial, and BBB-penetrating properties. Fluorescence imaging and brain tissue section analysis in mice demonstrated that CD-Ce6 exhibited strong accumulation in the brain regions after systemic administration, in contrast to free Ce6, which showed negligible brain penetration (Figure 9B-D). This confirms that the CD component effectively enhances BBB penetration, likely because of its small size, surface properties, and biointeractions, making CD-Ce6 a promising platform for brain-targeted phototherapeutic interventions 124.

### 4.5. Sonodynamic therapy

**Figure 10:**
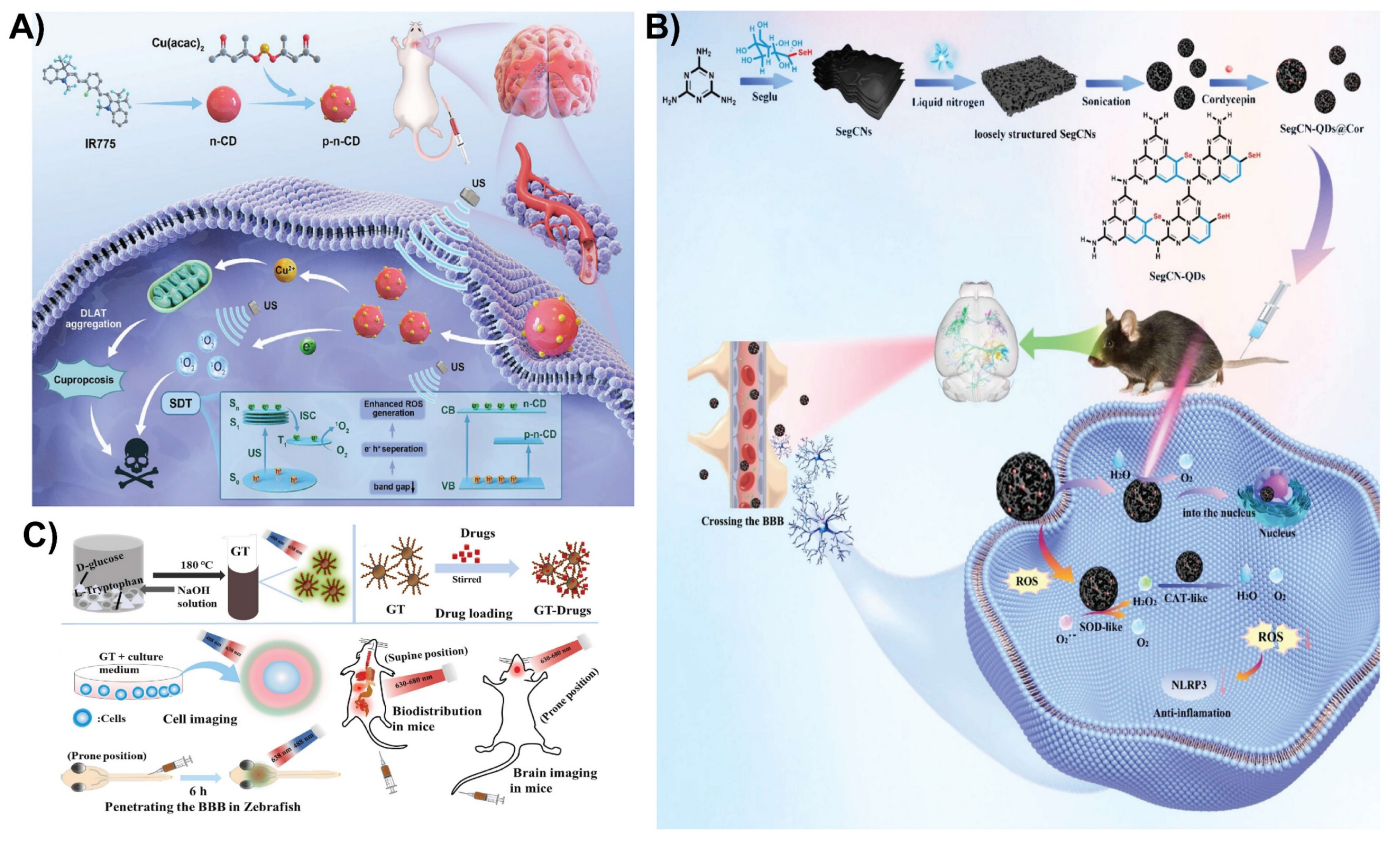
A) Schematic illustration of Cu-doped carbon dots (Cu-CDs) featuring a long-lived triplet excited state (T₁) and a p-n-type heterojunction, designed for brain imaging and sonodynamic cancer therapy. Reproduced with permission. 126 Copyright 2024, Wiley-VCH. B) Schematic illustration of SegCN-QDs@Cor-mediated microglia-targeted delivery to the mouse substantia nigra and striatum with 808 nm light assistance, along with its molecular mechanism. Reproduced with permission. 136 Copyright 2024, Elsevier. C) Overall schematic of the chemical synthesis of the multi-emission fluorescence platform and its biomedical applications. Reproduced with permission from 142, copyright 2023, Elsevier.

Sonodynamic therapy is a noninvasive treatment that uses low-intensity ultrasound to activate sonosensitizers, generating ROS that induce tumor cell apoptosis. It is effective for deep-seated tumors, such as glioblastomas, owing to its tissue-penetrating ability 125. CDs are emerging as promising nanomaterials for sonodynamic therapy in the treatment of brain diseases, particularly glioblastomas. Recently, Cheng *et al.* demonstrated a novel sonosensitizer using copper-doped CDs (Cu-CDs) for sonodynamic therapy of glioblastoma multiforme. The Cu-CDs were transformed into p-n-type semiconductors with a 1.58 eV bandgap and a prolonged 10.7 µs lifespan, enhancing ROS production by improving the electron-hole separation efficiency under low-intensity ultrasound. This sonodynamic effect, combined with the induction of cuproptosis (copper-mediated cell death) via the upregulation of dihydrosulfanyl transacetylase, synergistically killed glioblastoma multiforme cells (Figure 10A). Cu-CDs demonstrate excellent BBB permeability and potent antitumor activity, inhibiting glioblastoma multiforme tumor growth and increasing survival rates in mouse models, offering a promising approach for noninvasive glioblastoma multiforme treatment with high safety and efficacy 126. In another recent report, rose bengal (RB)-modified CDs functionalized with RGD peptides, termed RB-CDs@RGD, selectively targeted integrin αvβ3 receptors that are highly expressed on glioma cells, facilitating their accumulation in brain tumors. Upon ultrasound activation during sonodynamic therapy, these nanoplatforms produce ROS, triggering oxidative stress-induced apoptosis of glioma cells. This approach offers a noninvasive treatment modality with deep-tissue penetration and high therapeutic efficacy. *In vivo* experiments revealed potent antitumor activity and excellent biocompatibility. In addition, the intrinsic fluorescence of RB-CDs@RGD supports fluorescence-guided surgery, accurately delineating tumor margins and reducing the risk of recurrence 127. In this modality, CDs absorb acoustic energy and convert it into chemical energy to produce singlet oxygen (^1^O₂), hydroxyl radicals (•OH), and other ROS. The presence of functional groups on CDs, along with doping elements such as nitrogen, sulfur, and metals, can significantly enhance ROS production. Combined with other therapies, CD-based sonodynamic therapy offers a noninvasive method to penetrate deep brain tissues, overcoming the depth limitations associated with photodynamic therapy.

### 4.6. Antioxidant and anti-inflammatory

CDs with antioxidants and anti-inflammatory properties hold significant promise for treating CNS diseases characterized by oxidative stress and chronic neuroinflammation 128. In conditions such as Alzheimer's disease, Parkinson's disease, traumatic brain injury, ischemic stroke, and multiple sclerosis, excessive production of ROS leads to mitochondrial dysfunction, lipid peroxidation, DNA damage, and neuronal apoptosis 129-131. CDs, particularly those doped with heteroatoms (e.g., nitrogen, sulfur, and phosphorus) or derived from antioxidant-rich precursors, exhibit strong free radical scavenging capabilities because of their abundant surface functional groups (e.g., -OH, -COOH, and -NH_2_) 132. These CDs can directly neutralize ROS and modulate redox balance within neural tissues. In parallel, CDs exert anti-inflammatory effects by downregulating pro-inflammatory cytokines (e.g., TNF-α, IL-1β, and IL-6) and inhibiting the activation of microglia and astrocytes, which are central mediators of neuroinflammation 133. Moreover, CDs can suppress the NF-κB and MAPK signaling pathways, which are commonly overactivated during CNS inflammation 134. Recently, CDs have been reported to enhance the delivery and efficacy of epigallocatechin-3-gallate (EGCG), a natural antioxidant, by improving its stability and brain penetration. In the neural system, EGCG-CDs act as antioxidants by scavenging ROS and as anti-inflammatory agents by inhibiting the NLRP3 inflammasome, a key driver of neuroinflammation. This dual action protects neurons, reduces oxidative stress, and suppresses inflammatory cytokines, making EGCG-CDs effective for treating brain injuries and neurodegenerative conditions 135. Se-doped CDs (Se-CDs) act as artificial nanozymes that mimic the activities of superoxide dismutase and catalase, which are key antioxidant enzymes. In neural systems, especially under Parkinson's disease-like conditions, these nanozymes actively scavenge ROS and mitigate oxidative stress. In addition, they inhibit the NF-κB/NLRP3 inflammasome signaling pathway, which is a critical mediator of neuroinflammation. This combined action reduced both ROS accumulation and inflammatory cytokine production, protecting dopaminergic neurons from degeneration and supporting the recovery of neurological function (Figure 10B). NIR activation further enhances the therapeutic efficacy by boosting ROS clearance in the deep brain tissues 136. Yang *et al.* engineered amine-functionalized aspirin-derived CDs with strong neuroprotective properties to address oxidative stress and neuroinflammation. These CDs function as efficient iron chelators, eliminating excess iron that contributes to ROS production after intracerebral hemorrhage, and act as antioxidants to directly scavenge ROS. Their application significantly reduced iron accumulation in the brain, alleviated meningeal inflammation, and enhanced neurological recovery in mice, underscoring their potential as a dual-function therapeutic approach for brain injury 137. More recently, Wang *et al.* reported a rational co-assembly strategy using L-glutamate-derived CDs (LCDs) and ferulic acid (FA) to form multifunctional nanoassemblies (FA-LCDs). Notably, the enhanced BBB transport of FA-LCDs was attributed to their physicochemical properties, including ultrasmall size and surface chemistry, rather than a receptor-targeting mechanism. Once across the BBB, the FA-LCDs showed specific affinity interactions with Aβ species, inhibiting Aβ fibrillation, disassembling existing aggregates, and clearing excessive ROS. These multifunctional activities alleviated Aβ burden, oxidative stress, neuronal damage, and cognitive deficits in Alzheimer's disease mouse models, highlighting the therapeutic potential of engineered CDs for CNS disorders 138.

When functionalized with brain-targeting ligands, coated with membrane-like biological species, or rationally designed, CDs can efficiently cross the BBB and accumulate in diseased brain areas. Targeted delivery enhances therapeutic efficacy and reduces systemic side effects. Antioxidant and anti-inflammatory CDs offer a multifunctional nanoplatform for neuroprotection, capable of mitigating oxidative damage, restoring neuronal homeostasis, and modulating immune responses in various CNS pathologies.

### 4.7. Imaging-guided therapeutics

Imaging-guided therapeutics represent a transformative approach to treat CNS diseases, enabling real-time visualization, precise drug delivery, and monitoring of therapeutic efficacy in complex brain environments 11. CDs are ideal candidates for theranostic systems because of their intrinsic photoluminescence, biocompatibility, and modifiability 139. When designed for dual or multimodal imaging, such as fluorescence, photoacoustic, or MR, CD-based nanoplatforms can be used to track biodistribution and accumulation at target sites (e.g., gliomas and Alzheimer's plaques) and to monitor pathological changes during therapy 121. For instance, CDs can be conjugated with targeting ligands (e.g., TfR, folic acid, and peptides) or cloaked in biomimetic membranes to cross the BBB and selectively accumulate in diseased areas 108. Upon reaching the target, these nanosystems can deliver drugs or genes or induce therapeutic effects via photothermal or photodynamic therapy with laser or NIR light activation, guided by imaging feedback 140. This allows visualization of the lesion, confirms nanocarrier accumulation, and precisely activates therapy at the desired location, minimizing damage to healthy tissues 141. In diseases such as glioblastoma, stroke, and neurodegenerative disorders, imaging-guided CD-based therapeutics improve treatment precision, reduce systemic toxicity, and allow personalized and timely interventions by integrating diagnostics and therapy into a single, intelligent nanoplatform. Yang *et al.* synthesized CDs from endogenous nutrient precursors that exhibited both visible- and NIR fluorescence. These ultrasmall CDs (<5 nm) can effectively cross the BBB through size-dependent passive diffusion. Once in the brain, NIR fluorescence enables deep tissue bioimaging, and NIR laser irradiation activates the CDs for photothermal therapy. Upon NIR exposure, the CDs convert light energy into localized heat, offering a minimally invasive therapeutic approach to damage pathological tissues, such as gliomas, without harming the surrounding healthy cells (Figure 10C). The dual functionality of noninvasive imaging and NIR-triggered therapy makes these CDs promising tools for both the diagnosis and treatment of CNS diseases 142. Highly crystalline multicolor CDs (HCCDs) have been effectively applied in dual-modal imaging-guided photothermal therapy for gliomas. These ultrasmall CDs (~6-8 nm) exhibit bright multicolor fluorescence and excellent photothermal conversion efficiencies under NIR light. Their crystalline structures enhance stability and light absorption, allowing them to generate localized heat upon NIR exposure. After systemic administration in mice bearing intracranial U87 glioma tumors, HCCDs successfully crossed the BBB and selectively accumulated in tumor tissues. Real-time tumor imaging was achieved using both fluorescence and photoacoustic modalities, guiding precise NIR laser irradiation. Upon laser exposure, localized heating leads to effective tumor cell ablation with minimal damage to the surrounding healthy brain tissue. This approach demonstrates high therapeutic efficacy, minimal toxicity, and potential for clinical translation in the treatment of brain cancers such as glioma 121. In another report, gadolinium-doped CDs (Gd-CDs) were used as multimodal agents for photothermal therapy and tumor monitoring in brain cancer. These biocompatible CDs absorbed NIR light and generated heat to ablate glioblastoma cells via photothermal therapy. Their gadolinium content enhances MRI contrast for tumor visualization, whereas their fluorescence enables optical imaging. Small-sized Gd-CDs penetrate the BBB and achieve effective tumor reduction *in vivo* with low toxicity, making them promising theranostic platforms for brain cancer treatment and monitoring 143.

**Figure 11:**
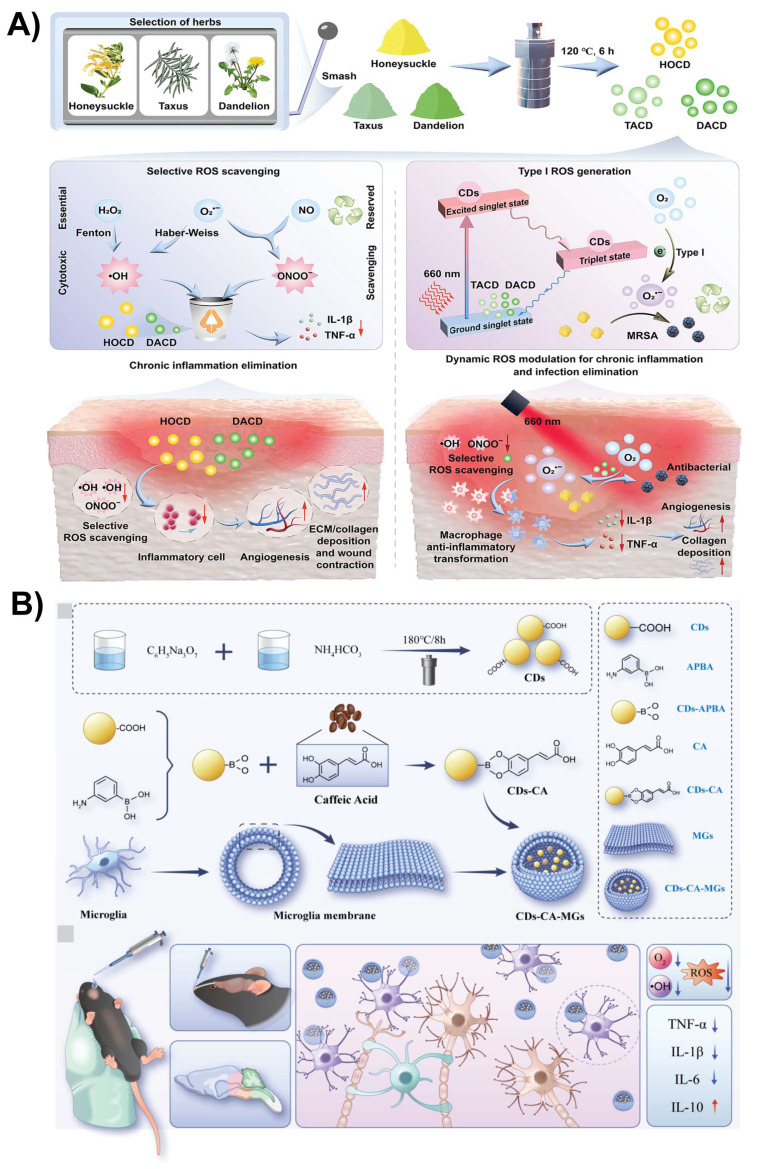
A) Schematic diagram of the preparation of three herbal CDs and their applications as dynamic ROS nanomodulators for anti-inflammatory and antibacterial purposes. Reproduced with permission from 128, copyright 2024, American Chemical Society. B) Preparation process and action mechanism of caffeic acid-CDs coated with microglia membrane (CDs-CA-MGs). Reproduced with permission from 133, copyright 2024, Springer Nature.

CDs represent a versatile class of nanotheranostic agents for CNS pathologies that integrate multimodal diagnostic capabilities with targeted therapy delivery. Their intrinsic photoluminescence facilitates high-resolution, non-invasive neuroimaging, while their tunable physicochemical properties and capacity for BBB translocation permit the precise delivery of pharmacological, genetic, or molecular payloads 11, 86. Coupling real-time imaging feedback with therapeutic activation allows spatiotemporal control of photothermal, photodynamic, sonodynamic, chemotherapeutic, or combinatorial interventions, maximizing on-target efficacy and minimizing collateral damage. Preclinical investigations have demonstrated the utility of CDs as both intrinsic and vectorized photo/sonosensitizers in gliomas and other brain tumor models, as well as their capacity to elicit photothermal therapy, ROS-mediated oxidative stress, antioxidant modulation, and anti-inflammatory responses (Figure 11A and B) 128, 133. These multifunctional attributes make CDs a next-generation platform for precision neurotherapeutics.

Owing to their ultrasmall size, surface chemistry, and BBB penetration ability, CDs can interact with key components of the tumor microenvironment and modulate immune responses through both direct and indirect mechanisms. Functionalized CDs have shown to influence macrophage and microglial polarizations, promoting a shift from immunosuppressive (M2-type) phenotypes toward pro-inflammatory, anti-tumor (M1-type) states, thereby enhancing local immune activation. Furthermore, CD-based nanoplatforms could facilitate antigen presentation by dendritic cells and improve T-cell priming via delivery of immunogenic cargos or adjuvants, conceptually aligning with established cancer immunotherapy strategies 144. For example, Hu *et al.* 133 functionalized CDs with bioactive molecules, which exhibited immunomodulatory effects in a brain disease model by attenuating neuroinflammation rather than directly activating adaptive immunity. The formulated CDs penetrated the BBB and reduced pro-inflammatory cytokines TNF-α, IL-1β, and IL-6 in the brain tissue, suggesting pathological immune activation (Figure 11B). This was accompanied by modulation of microglial activation and reduction of oxidative stress-driven inflammatory signaling. These findings suggest that, while current CD-based approaches function primarily through innate immune modulation rather than classical immune checkpoint or T-cell-based immunotherapy, they provide a mechanistic foundation for developing CD nanoplatforms that integrate neuroimmune regulation into future CNS immunotherapeutic strategies.

To be effective in brain theranostics, CDs must meet several design requirements, which are summarized in Table 4. By fulfilling these design criteria, CDs have the potential to be used as next-generation nanotheranostic agents for the personalized diagnosis and treatment of complex CNS diseases.

## 5. Current limitations and future perspectives

In general, the translation of CDs to clinical settings is hindered by several critical interrelated obstacles, including a lack of synthesis standardizations, physicochemical instability, unclear biological fate, and incomplete safety evaluation. A major challenge is the lack of scalable and reproducible manufacturing protocols. Batch-to-batch variability in precursor composition, reaction condition, and post-synthesis processing often results in heterogeneous size distributions and inconsistent photoluminescence properties, which significantly compromise reproducibility and regulatory acceptance.

Surface functionalization, while essential for targeting and drug loading, can introduce additional complexity. In many cases, adding targeting ligands or functional groups induces aggregation, causes surface oxidation, and deteriorates the emission profile. Furthermore, weakly bound surface moieties may detach from the carbon core before reaching the target, reducing the therapeutic efficacy of CDs. Although CDs have been reported to have lower toxicity compared with semiconductor quantum dots, information regarding their long-term effects, pharmacokinetics, and body clearance remains inadequate, particularly under repeated dosing and chronic administration. The absence of standardized metrics for dimensional characterization (hydrodynamic vs. core size), surface chemistry quantification, and photophysical property assessment contribute to inconsistent reporting and complicated data interpretation across studies. The *in vivo* fate of CDs, including renal versus hepatobiliary clearance pathways, biodegradation, organ accumulation, immunogenicity, and potential genotoxicity, requires comprehensive long-term evaluation. Even with high-affinity targeting ligands, only a small portion of systemically administered CDs reaches therapeutically relevant concentrations in the brain parenchyma, with considerable off-target accumulation in clearance organs such as the liver and spleen.

In contrast, several FDA-approved nanomedicines, such as liposomal formulations and polymer-based nanoparticles, have well-defined physicochemical specifications, pharmacokinetic parameters, clearance pathways, and safety profiles established through rigorous preclinical and clinical evaluation. Compared with clinically approved platforms, CDs lack standardized and comprehensive pharmacokinetic parameters, such as quantitative brain-to-blood concentration ratios, administered dose accumulated in the brain, organ-level biodistribution profiles, clearance pathways, and long-term immunotoxicity assessments. Therefore, direct comparison with established nanocarriers, as well as among different CD formulations, is difficult, hindering the rational positioning of CDs within the existing nanotherapeutics landscape.

Live cell-based delivery has recently emerged as a promising approach for brain targeting. Integrating CDs with living carriers, such as immune or stem cells, may synergistically combine active homing and BBB-crossing capabilities with CD-based theranostics, although this strategy remains at an early proof-of-concept stage and requires further investigation to address biosafety and translational challenges.

To advance clinical translation, future research should prioritize the rational design of ultrasmall (<10 nm) CDs with engineered NIR emission for deep-tissue imaging and rapid renal clearance. Developing stimuli-responsive nanoplatforms such as pH-, enzyme-, ROS-, or ultrasound-responsive linkers could enable spatiotemporally controlled drug release while minimizing off-target effects. Equally important is the adoption of harmonized characterization standards. Comprehensive toxicological studies, including repeat-dose, reproductive, and genotoxic endpoints, coupled with biodistribution and clearance data, are essential for accelerating clinical translation. Systematic comparison of CDs with clinically approved nanomedicines, using consistent dosing, administration routes, and evaluation endpoints, will be essential to assess their translational potential and to position CDs within the broader landscape of clinically viable nanotherapeutics.

## 6. Summary

This review summarizes recent advances in the design of CDs that cross the BBB for targeting, diagnosis, and treatment of CNS diseases, or for theranostics. To facilitate effective BBB crossing, CDs can be functionalized with targeting ligands, coated with polymers, or modified with biomimetic membranes, exosomes, or other ligands, allowing them to target diseased cells or biomarkers. The collective engineering of these functional groups supports precise control over the surface charge of CDs, which is pivotal for stability against aggregation in physiological solutions and for directing cell-specific targeting. Surface charges affect how CDs interact with cell membranes, dictating internalization pathways and ultimately their intracellular fate. CDs have emerged as highly promising nanomaterials for the diagnosis and treatment of neurodegenerative diseases and brain cancers owing to their unique physicochemical and biological properties. Their ultrasmall size, tunable surface functionalities, excellent photoluminescence, and biocompatibility enable them to cross the BBB efficiently and selectively accumulate in brain tissue. CDs can serve as multimodal imaging agents that offer fluorescence, MRI, or photoacoustic imaging, facilitating early detection and real-time monitoring of brain diseases. In addition, they can be engineered to deliver drugs, genes, or therapeutic agents specifically to diseased areas, reducing off-target effects. Their antioxidants, anti-inflammatory, photothermal, and photodynamic therapeutic properties further enhance their potential as theranostic agents. These attributes collectively position CDs as a transformative platform for the integrated diagnosis and targeted therapy of complex CNS disorders. Hybrid nanotherapeutic strategies that integrate CDs carriers with targeting peptides, receptor-specific ligands, PEG coatings, or stimuli-responsive modalities (light, ultrasound, and ROS) have demonstrated enhanced BBB penetration and disease-specific payload release. Biomimetic cloaking, such as macrophage membrane coating, introduces endothelial adhesion proteins (e.g. vascular cell adhesion molecule-1 ligands) that facilitate the selective recognition of the inflamed brain vasculature, further improving targeting precision. Despite these advances, significant translation barriers exist. CDs synthesized from diverse precursors and fabrication routes exhibit substantial heterogeneity in terms of particle size, surface functionality, quantum yield, and residual impurities, hindering their cross-study comparability and complicating regulatory validation.

Overall, the rapid progress in CD engineering strategies has established a strong foundation for their application in CNS targeting, diagnosis, and therapy. Continued refinement in synthesis control and surface design is expected to improve reproducibility and translational robustness, reinforcing the potential of CDs as versatile and clinically relevant nanoplatforms for brain diseases.

## Figures and Tables

**Table 1:** Comparison of the advantages and limitations of various nanocarriers: CDs, liposomes, polymer nanoparticles, inorganic nanoparticles, and exosomes, focusing on CNS delivery and translational feasibility

Nanocarrier type	Advantages	Limitations	CNS delivery relevance and translational potential	Ref.
CDs	Ultra small size allowing BBB penetration High biocompatibility and low toxicity Intrinsic fluorescence useful for imaging Functional groups allow conjugation of various drugs and ligands	Aggregation and reduced stability in biological media Incomplete *in vivo* safety and clearance data Lack of clinical validation.	CDs can cross the BBB in animal models, but their CNS translation is limited by inadequate pharmacokinetic, neurotoxicity, biodegradation data, and lack of regulatory precedent.	11, 86, 100, 110
Liposomes	Excellent biocompatibility and safety profile Able to encapsulate both hydrophilic and hydrophobic drugs Several FDA-approved liposomal drugs show clinical success.	Poor stability and payload leakage Limited BBB penetration without modification Rapid reticuloendothelial system clearance and immune activation. High cost and cold-chain-dependent manufacturing.	Liposomes have poor inherent BBB permeability and require PEGylation or ligand targeting for CNS delivery, increasing complexity and limiting clinical translation.	92, 94, 145
Polymer nanoparticles	Tunable size, degradability, and release profiles Cargo protection and extended circulation Potential immunogenicity	Complex synthesis and limited reproducibility Limited BBB permeability due to size, requiring active transport	Certain polymer nanoparticles can cross the BBB via ligand- or transport-mediated mechanisms, but CNS clinical translation remains challenging.	146, 147
Inorganic nanoparticles	Excellent for imaging (optical/magnetic) and multifunctional systems Certain nanoparticles, like gold/silica, can be engineered to facilitate BBB transport.	Potential organ accumulation and toxicity Agglomeration and rapid immune clearance Unclear long-term neural effects	Although functionalized inorganic nanoparticles can cross the BBB, concerns over chronic accumulation and neurotoxicity limit their long-term CNS use.	13, 94, 148
Exosomes	Highly biocompatible, cell-derived vesicles with targeting potential Low immunogenicity as endogenous communication carriers	Non-standardized isolation and purification Storage and stability limitations Biological heterogeneity complicates dosing and regulation.	Show promise for CNS delivery due to their BBB-crossing ability, but clinical translation is limited by issues related to scale-up, batch consistency, regulation, and dose control.	109, 149, 150

BBB: blood-brain barrier; CDs: carbon dots; CNS: central nervous disease; FDA: food and drug administration.

**Table 2:** Summary of various modification strategies of CDs for BBB crossing in CNS disease diagnostics and theranostics

Functionalization strategies		Size of CDs (nm)	Targeting mechanisms	Disease model	Applications	Ref.
Ligand functionalization	TfR-conjugation of CDs	2-6	TfR-mediated endocytosis	Glioma	Imaging and drug delivery	116
	Angiopep-2 decoration of CDs	4	LRP1-mediated transcytosis	Glioma	Glioma targeting with imaging capability	151
	Glucose-derived CDs	3.77	GLUT1 transporter-mediated transport	Neurodegeneration & glioma	BBB crossing and neuronal imaging	86
	Tryptophan-derived CDs	4-7	LAT1 transporter-mediated transport	Neurodegeneration & glioma	BBB crossing and glioblastoma cell uptake	152
	IL-6 peptide-functionalized polymer-coated CDs	5-10	IL-6 receptor targeting	Glioma	Glioma targeting, pH-triggered drug release, and imaging	99
	Aptamer-functionalized CDs	5	Aβ-targeting *via* aptamer and photomodulation	Alzheimer's disease	Target Aβ, red-light-induced denaturation, and plaque reduction	153
Polymer coating strategies	Ginkgolide B-loaded carbonized CDs	5	Delivery through a polymer matrix	Ischemic stroke	Decrease infarct size, reduce neuronal apoptosis and inflammation	154
	Metformin CDs	2.95	Passive diffusion	Alzheimer's disease	Promote neurogenesis, reduce Aβ deposition, and provide neuroprotection	155
	PEG- Lf Coated CDs	4.5	Lf receptor-mediated transportation	Parkinson's disease	Nitric oxide-releasing CDs reduce ROS, chelate Fe²⁺, and suppress α-synuclein aggregation	103
	Polydopamine-coated CDs in macrophage-derived exosomes	3.15	Exosome-mediated delivery	Parkinson's disease	Reduce oxidative stress, alleviate symptoms through anti-inflammatory/photothermal therapy	156
	Triple-functionalized HSA-conjugated CDs (TfR + RVG29 + Angiopep-2)	2-4	RMT *via* three ligands	Alzheimer's disease	Detect Aβ aggregates, prevent aggregation, scavenge ROS, enable *in vivo* imaging	23
Biomimetic membrane/ exosome coating	Macrophage-exosome-coated CDs	2	Exosome-mediated homing	Glioma	Precise boron delivery for BNCT, *in vivo* survival	106
	Exosome-coated polydopamine CDs	2-4	Biomimetic membrane homing and receptor-mediated uptake	Parkinson's disease	BBB crossing, target neuroinflammation to restore injured neurons by eliminating oxidative stress	107
	Microglia membrane-wrapped caffeic acid-CDs	5	Biomimetic homing and nasal route to bypass the BBB	Alzheimer's disease	Microglial membrane enhances brain targeting, reduces neuroinflammation, and improves cognition	133
Other modification strategies	Vitamin C-derived CDs	4	Passive diffusion	Alzheimer's disease	Inhibit Aβ aggregation, ROS scavenging	129
	Aspartic acid-derived CDs	2.28	Self-targeting via glioma affinity	Brain cancer	Intrinsic glioma targeting and imaging	108
	Quercetin-derived red-emission CDs (R-CD-75)	3.66	Passive diffusion, hydrophobic/π-π/H-bond interactions	Alzheimer's disease	Inhibit Aβ aggregation, red fluorescence imaging	157
	Memantine-loaded CDs	2.2	Passive diffusion	Alzheimer's (tau aggregation)	Deliver memantine and inhibit tau aggregation	118
	Crinis Carbonisatus-derived CDs	3.2-8.8	Passive diffusion	Ischemic stroke (cerebral I/R injury)	Reduce infarct volume, inflammation, and neuroprotection	158
	Fucoidan-derived CDs with sulfate groups	1.83	Negative-charge mediated transcytosis	Parkinson's disease	Antioxidant/anti-inflammatory effects	21
	CDs with amphiphilic surface	3	Passive diffusion	Alzheimer's disease	Inhibit APP & Aβ overexpression and allow BBB penetration	95
	Endogenous nutrient-based CDs	6.21	Passive diffusion	Glioma	Visible/NIR imaging and drug delivery	142

TfR: Transferrin, Aβ: β-amyloid, APP: Amyloid precursor protein, BNCT: Boron neutron capture therapy, GLUT1: Glucose transporter 1, IL-6: Interleukin-6, LAT1: L-type amino acid transporter 1, LRP1: Low-density lipoprotein receptor-related protein 1, RVG29: Rabies virus glycoprotein 29; PEG-Lf: polyethylene glycol Lactoferrin

**Table 3:** Summary of the main CNS diseases, their brain pathologies, their symptoms, and mechanisms of CDs in treatment

Representative CNS Disease	Targeted brain elements	Pathological roles	Mechanism of treatment with CDs
Alzheimer's disease	Microglia, Aβ receptors (RAGE, TREM2), Synapses 159	Neuroinflammation via microglial activation; Aβ plaque accumulation	Inhibit Aβ aggregation, reduce ROS, conjugate with drugs (e.g., memantine) for targeted delivery, and fluorescent imaging of Aβ plaques for early diagnosis 160, 161.
Parkinson's disease	Dopamine receptors (D1/D2), Microglia 162	Dopaminergic neuron degeneration, neuroinflammation	Scavenge ROS, promote neurodegeneration, deliver neuroprotective agents (e.g., dopamine precursors), fluorescent tracking of drug distribution, and imaging Lewy bodies 163.
Brain tumor (e.g., Glioblastoma)	TME, EGFR, BBB 164	Tumor growth, BBB disruption, resistance to therapy	Deliver chemotherapeutic drugs, induce photodynamic therapy via ROS generation, target tumor cells with conjugated drugs (e.g., doxorubicin), and enable fluorescent imaging for tumor localization and treatment monitoring 87, 165.
Traumatic brain injury	Tight junctions (occludin, claudins), ROS, Microglia 166	BBB disruption, oxidative damage, neuroinflammation	Scavenge ROS, reduce inflammation, promote neuronal repair, deliver anti-inflammatory or neuroprotective agents (e.g., minocycline), and use fluorescent imaging to monitor injury sites and enhance treatment efficacy 167, 168.
Ischemic stroke	Glutamate receptors (NMDA), ROS, Endothelial cells 169	Excitotoxicity, BBB breakdown, oxidative stress	Scavenge ROS, mitigate excitotoxicity, reduce inflammation, deliver thrombolytic or neuroprotective drugs (e.g., tissue plasminogen activator), and image ischemic areas for real-time monitoring of tissue damage and drug distribution 158, 170.
Depression	Serotonin receptors (5-HT1A/2A), BDNF 171	Neurotransmitter imbalance, neuronal atrophy	Modulate oxidative stress, enhance neurogenesis, reduce inflammation, deliver antidepressants (e.g., SSRIs like fluoxetine) to specific brain regions, imaging to study neurotransmitter pathways or monitor treatment response 130, 172.

BDNF: brain-derived neurotrophic factor, RAGE: receptor for advanced glycation end products, TREM2: triggering receptor expressed on myeloid cells 2, EGFR: epidermal growth factor receptor, ROS: reactive oxygen species, TME: tumor microenvironment, 5-HTA1A/2A: 5-hydroxytryptamine (serotonin) receptors 1A and 2A, NMDA: N-methyl-D-aspartate receptor.

**Table 4:** Summary of CDs for theranostic applications in brain diseases and their design requirements

Stages (↓)	Biological target /barrier	CD properties/modifications required	Predicted function	Ref.
Administration & circulation	Systemic circulation (IV injection)	High colloidal stability Long circulation time Biocompatibility & non-toxicity	Avoid immune clearance and reach brain vasculature	21, 173
BBB penetration	Tight junctions of endothelial cells	Less than 10 nm in size Surface functionalization with TfR, ApoE, TAT peptide, and exosome coating Neutral/positive surface charge	Transcytosis / receptor-mediated transport across the BBB	91, 95
Brain targeting	Neurons, glia, tumor cells	Ligand-receptor binding (e.g., EGFR, TfR) pH-responsive or ROS-triggered release Stimuli-responsive targeting	Accumulate at pathological sites (tumors, plaques)	116, 151, 152
Theranostic action	Cellular level	Fluorescence/MR imaging Photothermal therapy Photodynamic therapy Drug/gene delivery Antioxidant & anti-inflammatory activity	Combined diagnosis & treatment	11
Biodegradation & clearance	Metabolism and excretion	Biodegradable core Renal clearance (<5.5 nm)	Safe elimination from the body	174, 175

ApoE: Apolipoprotein E; BBB: Blood-brain barrier; CDs: carbon dots; EGFR: Epidermal Growth Factor Receptor; IV: intravenous; MRI: Magnetic resonance imaging; ROS: Reactive oxygen species; TAT: Trans-activating transcriptional activator; TfR: Transferrin Receptor.

## Abbreviations

5-HTA1A/2A: 5-hydroxytryptamine (serotonin) receptors 1A and 2A
AMT: adsorption-mediated transcytosis
APP: amyloid precursor protein
AFM: atomic force microscopy
Aβ: β-amyloid
BDNF: brain-derived neurotrophic factor
BBB: blood-brain barrier
BTB: blood-tumor barrier
BNCT: boron neutron capture therapy
^10^BCDs: boron-enriched CDs
CDs: carbon dots
CNS: central nervous system
Cur-CDs: curcumin-derived CDs
DOX: doxorubicin
EGFR: epidermal growth factor receptor
EGCG: epigallocatechin-3-gallate
FA: ferulic acid
FTIR: Fourier transform infrared spectroscopy
GLUT1: glucose transporter 1
HCCDs: highly crystalline multicolor CDs
HR-TEM: high-resolution transmission electron microscopy
IL-6: interleukin-6
LAT1: L-type amino acid transporter 1
LCDs: L-glutamate-derived CDs
LRP1: low-density lipoprotein receptor-related protein 1
LAT1: L-type amino acid transporter 1 (LAT1)
NIR: near infrared
N-CDs: nitrogen-doped CDs (N-CDs)
NMDA: N-methyl-D-aspartate receptor
PEG-Lf: polyethylene glycol lactoferrin
PCDs: polydopamine-CDs
PEG: polyethylene glycol
PEI: polyethyleneimine
RVG29: rabies virus glycoprotein 29
RAGE: receptor for advanced glycation end products
RMT: receptor-mediated transcytosis
ROS: reactive oxygen species
TfR: transferrin
TfR-CDs: TfR-conjugated CDs
TME: tumor microenvironment
TREM2: triggering receptor expressed on myeloid cells 2
XPS: X-ray photoelectron spectroscopy
